# Metabolic patterns in brain ^18^F-fluorodeoxyglucose PET relate to aetiology in paediatric dystonia

**DOI:** 10.1093/brain/awac439

**Published:** 2022-11-29

**Authors:** Stavros Tsagkaris, Eric K C Yau, Verity McClelland, Apostolos Papandreou, Ata Siddiqui, Daniel E Lumsden, Margaret Kaminska, Eric Guedj, Alexander Hammers, Jean-Pierre Lin

**Affiliations:** Children’s Neurosciences, Complex Motor Disorders Service (CMDS), Evelina London Children's Hospital, Guy's and St Thomas’ NHS Foundation Trust (GSTT), London SE1 7EH, UK; King’s College London & Guy’s and St Thomas’ PET Centre, Division of Biomedical Engineering and Imaging Sciences, King’s College London, London SE1 7EH, UK; Department of Paediatrics & Adolescent Medicine, Princess Margaret Hospital, Kowloon, Hong Kong; Children’s Neurosciences, Complex Motor Disorders Service (CMDS), Evelina London Children's Hospital, Guy's and St Thomas’ NHS Foundation Trust (GSTT), London SE1 7EH, UK; Department of Basic and Clinical Neuroscience, Institute of Psychiatry, Psychology and Neuroscience, King’s College London, London SE5 8AF, UK; Children’s Neurosciences, Complex Motor Disorders Service (CMDS), Evelina London Children's Hospital, Guy's and St Thomas’ NHS Foundation Trust (GSTT), London SE1 7EH, UK; Developmental Neurosciences, Zayed Centre for Research into Rare Disease in Children, University College London Great Ormond Street Institute of Child Health, London WC1N 1DZ, UK; Neuroradiology Department, Evelina London Children's Hospital, Guy's and St Thomas’ NHS Foundation Trust (GSTT), London SE1 7EH, UK; Children’s Neurosciences, Complex Motor Disorders Service (CMDS), Evelina London Children's Hospital, Guy's and St Thomas’ NHS Foundation Trust (GSTT), London SE1 7EH, UK; Perinatal Imaging, Division of Biomedical Engineering and Imaging Sciences, King’s College London, London SE1 7EH, UK; Children’s Neurosciences, Complex Motor Disorders Service (CMDS), Evelina London Children's Hospital, Guy's and St Thomas’ NHS Foundation Trust (GSTT), London SE1 7EH, UK; CERIMED, Nuclear Medicine Department, Aix Marseille Universite, APHM, CNRS, Centrale Marseille, Institut Fresnel, Timone Hospital, 13397 Marseille, France; King’s College London & Guy’s and St Thomas’ PET Centre, Division of Biomedical Engineering and Imaging Sciences, King’s College London, London SE1 7EH, UK; Children’s Neurosciences, Complex Motor Disorders Service (CMDS), Evelina London Children's Hospital, Guy's and St Thomas’ NHS Foundation Trust (GSTT), London SE1 7EH, UK; Women and Children’s Health Institute Faculty of Life Sciences & Medicine, Kings Health Partners, King’s College London, London SE1 7EH, UK

**Keywords:** inherited childhood dystonia, dystonic cerebral palsy, PET functional imaging

## Abstract

There is a lack of imaging markers revealing the functional characteristics of different brain regions in paediatric dystonia. In this observational study, we assessed the utility of [^18^F]2-fluoro-2-deoxy-D-glucose (FDG)-PET in understanding dystonia pathophysiology by revealing specific resting awake brain glucose metabolism patterns in different childhood dystonia subgroups. PET scans from 267 children with dystonia being evaluated for possible deep brain stimulation surgery between September 2007 and February 2018 at Evelina London Children’s Hospital (ELCH), UK, were examined. Scans without gross anatomical abnormality (e.g. large cysts, significant ventriculomegaly; *n* = 240) were analysed with Statistical Parametric Mapping (SPM12). Glucose metabolism patterns were examined in the 144/240 (60%) cases with the 10 commonest childhood-onset dystonias, focusing on nine anatomical regions. A group of 39 adult controls was used for comparisons. The genetic dystonias were associated with the following genes: *TOR1A*, *THAP1*, *SGCE*, *KMT2B*, *HPRT1* (Lesch Nyhan disease), *PANK2* and *GCDH* (Glutaric Aciduria type 1). The acquired cerebral palsy (CP) cases were divided into those related to prematurity (CP-Preterm), neonatal jaundice/kernicterus (CP-Kernicterus) and hypoxic-ischaemic encephalopathy (CP-Term). Each dystonia subgroup had distinct patterns of altered FDG-PET uptake. Focal glucose hypometabolism of the pallidi, putamina or both, was the commonest finding, except in *PANK2*, where basal ganglia metabolism appeared normal. *HPRT1* uniquely showed glucose hypometabolism across all nine cerebral regions. Temporal lobe glucose hypometabolism was found in *KMT2B*, *HPRT1* and CP-Kernicterus. Frontal lobe hypometabolism was found in *SGCE*, *HPRT1* and *PANK2*. Thalamic and brainstem hypometabolism were seen only in *HPRT1*, CP-Preterm and CP-term dystonia cases. The combination of frontal and parietal lobe hypermetabolism was uniquely found in CP-term cases. *PANK2* cases showed a distinct combination of parietal hypermetabolism with cerebellar hypometabolism but intact putaminal-pallidal glucose metabolism. *HPRT1*, *PANK2*, CP-kernicterus and CP-preterm cases had cerebellar and insula glucose hypometabolism as well as parietal glucose hypermetabolism. The study findings offer insights into the pathophysiology of dystonia and support the network theory for dystonia pathogenesis. ‘Signature’ patterns for each dystonia subgroup could be a useful biomarker to guide differential diagnosis and inform personalized management strategies.

## Teaser

Tsagkaris *et al.* report that subgroups of patients with paediatric dystonia with different aetiologies show distinct patterns of ^18^F-FDG-PET metabolism that can be linked to the clinical signs in each group. These signature patterns could be useful imaging biomarkers to guide differential diagnosis and personalised management.

## Introduction

Dystonia has been operationally described as ‘a movement disorder characterized by sustained or intermittent muscle contractions causing abnormal, often repetitive movements, postures, or both. Dystonic movements are typically patterned, twisting, and may be tremulous. Dystonia is often initiated or worsened by voluntary action and associated with overflow muscle activation’.^1^

In contrast with adult-onset dystonias, childhood-onset dystonias are usually generalized, severe and of early onset; the most common aetiology is dystonic-dyskinetic cerebral palsy (CP) following perinatal brain injury, but primary genetic causes also exist. Paediatric dystonia is under-recognized,^2^ difficult to diagnose and classify,^3^ exerts a profound impact on children and their development^4^ and impairs growth with risk of musculoskeletal deformities in a period of maximal growth.^5^ Current pharmacological and surgical interventions often do not meet the needs of children with dystonia,^6^ especially as needs evolve with time.^7,8^ Further understanding of structural and functional characteristics of dystonia is needed to personalize care. Advanced therapeutics such as deep brain stimulation (DBS) provide new opportunities towards patient-specific improved outcomes. However, diagnostic markers for accurate and prompt diagnosis of dystonia subtypes, but also for informing personalized anti-dystonia management decisions, are often lacking.^9^

Dystonias are now widely seen as network disorders involving many brain regions, including the thalami, basal ganglia, sensorimotor cortex and cerebellum.^10^ Structural damage may be subtle, with no obvious brain lesions seen on MRI in many cases (including those of isolated genetic or idiopathic aetiology and a proportion of those with acquired dystonia, such as CP). Secondary structural re-organization arising from a life-long movement disorder with onset in early childhood cannot be detected with routine clinical brain imaging techniques.

However, functional abnormalities within the sensorimotor network exist in dystonia. Neurophysiological studies have revealed reduced inhibition throughout the nervous system,^11^ exaggerated cortical plasticity,^12^ distorted sensorimotor processing^13,14^ including abnormal sensorimotor neuronal connectivity^15–17^ and pathologically increased low-frequency neuronal oscillations within cortical-basal ganglia circuits.^18^ Whilst short latency cortical somatosensory evoked potentials (SEPs) are typically normal in isolated inherited/idiopathic dystonias, they may be abnormal in up to 47% of acquired dystonias like CP,^19^ including in a proportion of individuals with normal cranial MRI. In children with dystonia with normal SEPs, there is evidence of excessive theta-band synchronization and widespread activation across the sensorimotor network^20^ as well as impaired modulation of sensorimotor cortex mu activity, demonstrating a developmental abnormality of sensorimotor processing common to isolated inherited/idiopathic dystonias and dystonic CP.^21^

Moreover, functional imaging also demonstrates brain abnormalities in dystonia. Functional MRI studies have identified increased receptive fields for sensory representation of clinically affected areas in focal dystonia such as writer’s cramp^22^ as well as abnormal neuronal connectivity.^15,16^ However, these methods tend to be limited to patients with focal or mild dystonias, due to the difficulties obtaining image data without severe motion artefact in individuals with severe and/or generalized dystonias.

Resting dystonia is observed across the spectrum of aetiologies in childhood, either in awake sitting or lying position, but dystonia is abolished by sleep.^23^ Reduction in awake resting dystonia and improving awake sitting and lying comfort is frequently a DBS goal. The resting state also forms an integral component of the Burke-Fahn Marsden Dystonia Rating Scale-motor (BFMDRS-M).^24^ Therefore, in this study, we sought to establish a functional imaging correlate of childhood dystonia in the awake resting state; for this purpose, we used [^18^F]2-fluoro-2-deoxy-D-glucose (FDG)-PET, an exquisitely sensitive form of functional imaging.

FDG uptake correlates with synaptic density^25^ and activity,^26^ reflecting synaptic enrichment of mitochondria,^27^ where glucose is metabolized. FDG-PET has demonstrated abnormal brain metabolic activity in several types of inherited dystonia including *TOR1A*,^28^*THAP1* and *SGCE*^29^ in small numbers of middle-aged adults and also in paediatric dystonia due to *PANK2* neurodegeneration with brain iron accumulation (NBIA).^30^ However, reports of FDG-PET findings in other more recently described inherited dystonias and the commoner acquired dystonias are lacking. Here, we report FDG-PET brain imaging findings from a large cohort of children with dystonia of different aetiologies and compare awake resting regional glucose uptake patterns across genetic and acquired sub-groups.

## Materials and methods

A retrospective anonymized cohort study was performed, examining children with dystonia who were assessed for bilateral globus pallidus internus DBS surgery eligibility between September 2007 and February 2018 at Evelina London Children’s Hospital (ELCH), UK. The study was performed with institutional approval (Service Evaluation GSTT Reference 10 319). Entry criteria for this study were: (i) dystonia as the predominant movement disorder; and (ii) FDG-PET-CT brain scan availability.

Each patient was medically examined by a consultant paediatric neurologist (J.P.L., M.K., D.L.), who confirmed the diagnosis and dystonia classification. Routine baseline management of children with dystonia referred for possible DBS at ELCH-King’s College Hospital also initially involved a multi-disciplinary team (MDT): clinical assessment by a nurse specialist, physiotherapist, occupational therapist, speech and language therapist and clinical psychologist. Neurophysiological screening with somatosensory evoked potentials (SEPs) and central motor conduction times (CMCTs), DBS protocol MRI brain imaging under general anaesthesia (GA) and FDG-PET-CT under GA were also performed. Only patients deemed eligible for possible DBS surgery subsequently underwent the full multi-modal MDT assessment. The presence of significantly abnormal MRI brain excluded DBS surgery. More recently, McClelland *et al*.^19^ have shown that neurophysiological abnormalities such as abnormal CMCT and/or SEPs are associated with less benefit from DBS; therefore, such patients were less likely to proceed to work-up with PET imaging.

### FDG-PET-CT image acquisition

Prior to October 2013, all children underwent FDG-PET-CT imaging on a GE (GE Medical Systems) Discovery ST and a Discovery VCT scanner. Thereafter, scans were conducted using a GE Discovery 710 scanner at the King’s College London & Guy’s and St Thomas’ PET Centre. The FDG dose injected was scaled relative to a 250 MBq dose for a 70 kg adult as 250/70 × child’s weight (kg). FDG was injected after a 3-h fast and followed by a 30-min uptake period in a quiet room.^31^ Brief GA was then induced, only for the duration of the PET-CT image acquisition, to eliminate dystonia-related motion artefact during scanning. GA was initiated after the uptake period; therefore, FDG uptake reflected brain metabolism during wakefulness. The duration of the emission scan was 15 min. Reconstruction was via ordered subset expectation maximization (OSEM) as described previously.^30^ Each scan was anonymized and transferred to the university network for analysis.

### Control PET images

The control group comprised 39 healthy adult controls aged between 21 and 59 years (mean 40, SD 11). The scans were provided by the Department of Nuclear Medicine, Assistance Publique Hôpitaux de Marseille and were acquired after a 4–6 h fast on a GE Discovery ST PET-CT scanner over 15 min after bolus intravenous injection of 150 MBq of FDG and a 30-min uptake period (with eyes closed) and reconstructed via OSEM (ClinicalTrials reference: NCT00484523). These healthy subjects were free of neurological/psychiatric symptoms or antecedent diagnosis, had a normal neuropsychological evaluation, and a normal brain MRI. This control PET database was ethically approved by CPP Sud Mediterranée (2007-A00180-53).

### FDG-PET-CT image analysis

Statistical Parametric Mapping (SPM12; Wellcome Trust Centre for Neuroimaging, https://www.fil.ion.ucl.ac.uk/spm/) was used for analysis of the PET images.

A study-specific template in standard anatomical space (Montreal Neurological Institute, MNI, 152) was created using PET and MRI scans that were both reported as visually normal from 17 children. PET scans were co-registered (but not re-sliced) to MRI and the spatial transformation to MNI space derived from the MRIs using Unified Segmentation.^32^ PET scans were then averaged and smoothed with a Gaussian kernel (full-width-at-half-maximum 6 mm) to create a PET template. All patient and control images were then directly (PET-to-PET) spatially normalized to this template and smoothed with an 8 mm kernel.

### Statistical analysis

For all SPM analyses used in this study, a relative masking threshold of 0.8 was selected. This empirically excludes non-brain voxels from the analysis. The SPM standard mean voxel value was used for estimating the global metabolism value for each scan. Every image was scaled so that the mean global value was 100. Analysis of covariance was used as the normalization option. The patient’s age at the time the scan was performed was used as a linear co-variate. A visualization threshold of *P* < 0.001 was used. A cluster of voxels was considered statistically significant if *P* < 0.05, taking into consideration both effect size and spatial extent. Results were then displayed on the 97-region version of the maximum probability atlas derived from the Hammersmith Brain Atlas^33–35^ (available at www.brain-development.org/brain-atlases), based on manual delineations of all regions on MRI scans of 30 healthy controls.

### Data availability

Data are available from the corresponding author on reasonable request.

## Results

### Patient ascertainment

Two hundred and sixty seven children with FDG-PET scans were identified, 27 (10%) of whom were excluded from the study due to gross anatomical abnormalities (e.g. large cysts, significant ventriculomegaly), which would affect the reliability of the imaging analysis. The remaining 240/267 patients (90%) had suitable scans for quantitative analysis.

A total of 185/240 (77%) had a specific diagnosis of either a genetic or acquired form of dystonia at the time of the analysis. Namely, 128/240 (53%) patients had an acquired cause of dystonia, whereas 57/240 (24%) had isolated-inherited dystonia. Conversely, 55/240 (23%) were classified as isolated-idiopathic dystonia cases.

For the acquired dystonia cases, the CP cases were subdivided into those related to prematurity (CP-Preterm); those related to neonatal jaundice/kernicterus (CP-Kernicterus) and those born at term with a history of hypoxic-ischaemic encephalopathy (CP-Term).

The age at time of scan for the entire group varied from 1.3 to 20.6 years (mean age 10.5 years, SD 4.4).

**Figure 1:**
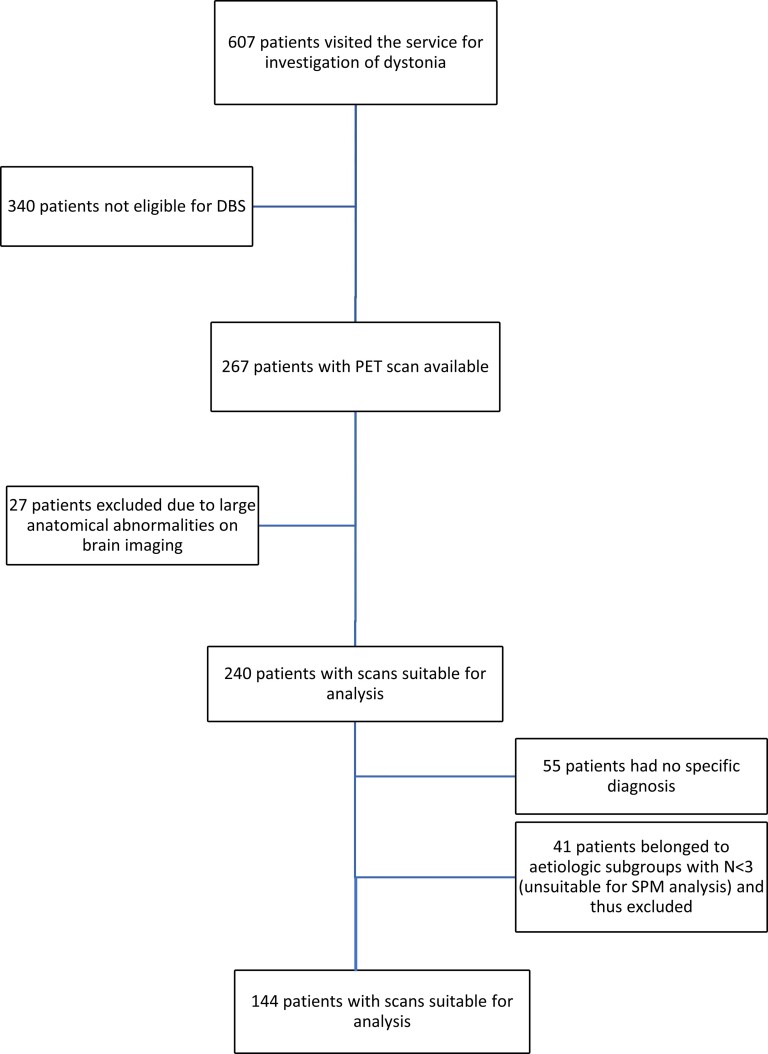
**Patient ascertainment flowchart**.

Overall, 144/185 (78%) (mean age 10.3 years, SD 4.0) patients belonged to an aetiologic subgroup with *n* ≥ 3 patients and were studied to investigate the regional brain glucose metabolism patterns in the different aetiologic subgroups of paediatric dystonia (Fig. 1).

Table 1 lists the demographic and severity characteristics of those 144 patients, which fell into the following aetiological groups based on the associated gene: *TOR1A*, *THAP1*, *SGCE*, *KMT2B*, *HPRT1*, *GCDH* (Glutaric aciduria type 1) or *PANK2*, and the three CP sub-groups listed above.

**Table 1:** Cohort demographics

Dystonia subgroup	Number of patients (*n*)	Age (mean ± SD)	BFMDRS-M (mean ± SD)
TOR1A (DYT1)	7	12.5 ± 2.9	40.3 ± 18.8
THAP1 (DYT6)	3	10.5 ± 3.0	70.0 ± 21.3
SGCE (DYT11)	6	11.6 ± 3.3	32.8 ± 9.2
KMT2B (DYT28)	7	10.6 ± 4.2	74.8 ± 6.0
HPRT1 (LND)	5	10.2 ± 4.4	82.8 ± 10.8
GCDH (GA-1)	9	10.4 ± 5.5	98.3 ± 17.2
PANK2 (PKAN)	14	10.1 ± 4.5	87.5 ± 19.4
CP-Kernicterus	13	9.6 ± 4.8	84.2 ± 23.4
CP-Preterm	35	8.1 ± 3.0	96.4 ± 13.0
CP-Term	45	11.8 ± 3.7	78.8 ± 21.2

Mean age at time of PET scan and BFMDRS-M score at baseline for the different aetiologic subgroups of dystonia. For the genetic aetiologies, the responsible gene name has been used throughout the text. Here, we also provide in parentheses for each of them other frequently used nomenclature involving the gene locus or the disease name, for ease of comparison with the wider literature. LND = Lesch–Nyhan disease; PKAN = pantothenate kinase-associated neurodegeneration.

### FDG-PET glucose metabolism in relation to dystonia aetiology

For aetiologic subgroups with a minimal sample size *n* ≥ 3 the main findings are described below and illustrated in Figs 2–6 (Supplementary Figs 1–10). Comparisons of relative hypo- and hypermetabolism across the groups are presented in Table 2. For ease of reference, we have combined all the figures into an A3 poster, which is available for printout in the Supplementary material.

**Figure 2:**
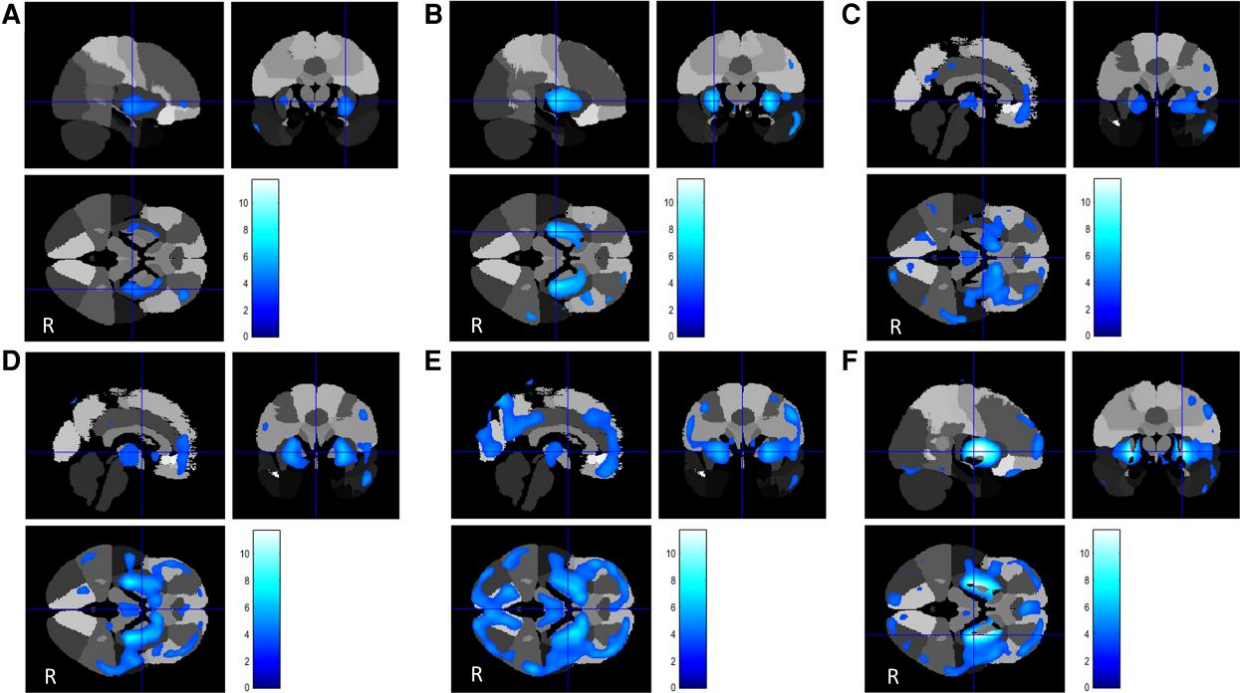
**FDG uptake compared to controls in inherited dystonias that only showed hypometabolism (excluding PANK2)**. Areas of relative regional hypometabolism are displayed in a blue-scale with brighter tones indicating higher t-scores. (**A**) TOR1A (*n* = 7): Putamen-frontal hypometabolism. (**B**) THAP1 (*n* = 3): Caudate-putaminal and right fronto-parietal hypometabolism. (**C**) SGCE (*n* = 6): Head of caudate, globus pallidus, inferior frontal lobe hypometabolism. (**D**) KMT2B (*n* = 7): Marked caudate-putaminal (striatal) and bi-frontal and bi-parietal hypometabolism. (**E**) HPRT1 (*n* = 5). Extreme caudate-putaminal-pallidal, medial thalamic, and pan-hemispheric and antero-superior cerebellar hypometabolism. (**F**) GCDH (*n* = 9). Relative regional hypometabolism in the posterior putamina and globi pallidi. All results are displayed on the 97-region version of the maximum probability atlas derived from the Hammers Atlas Database^33–35^ (www.brain-development.org/brain-atlases), which is a 3D maximum probability atlas of the human brain.

**Figure 3:**
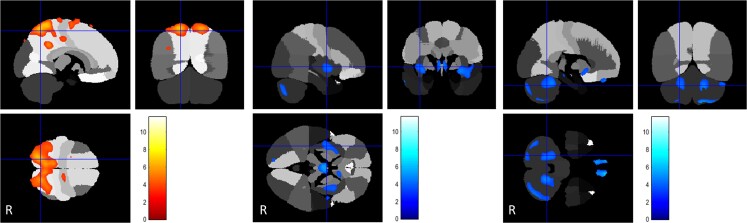
**PANK2**. FDG uptake in pantothenate kinase associated neurodegeneration cases (*n* = 14) compared to controls. *Left*: Areas of regional relative hypermetabolism seen in the superior parietal lobes and displayed in a yellow-scale with brighter tones indicating higher t-scores. *Middle* and *right*: Hypometabolic areas noted in the peri-insular cortex, cerebellar dentate nuclei and posterior inferior cerebellar cortex and displayed in a blue-scale with brighter tones indicating higher *t*-scores.

**Figure 4:**
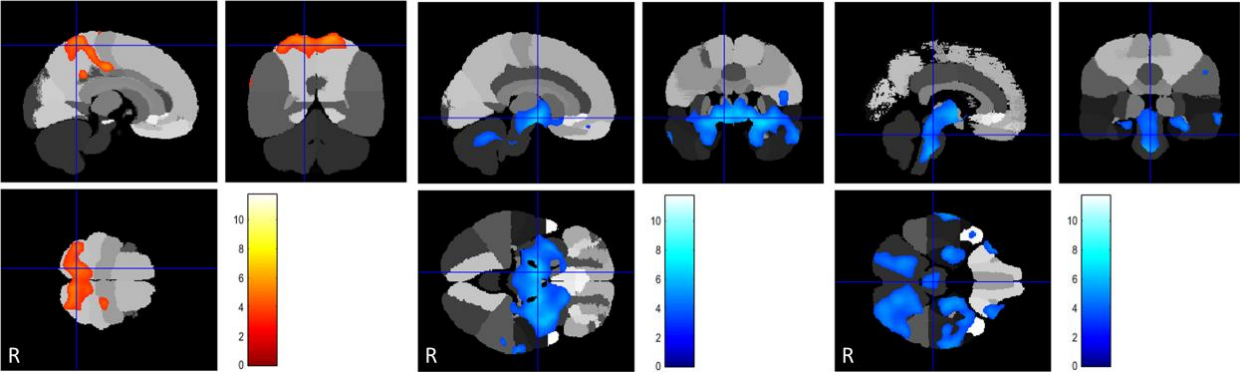
**FDG uptake in CP-kernicterus cases (*n* = 13) compared to controls**. *Left*: Relative hypermetabolism in superior parietal lobes displayed in a yellow-scale with brighter tones indicating higher *t*-scores. *Middle* and *right*: Areas of relative hypometabolism, including the antero-medial temporal cortex, globi pallidi, thalami, midbrain, pons, dentate-cortical cerebellum and peri-insular cortex, displayed in a blue-scale with brighter tones indicating higher *t*-scores.

**Figure 5:**
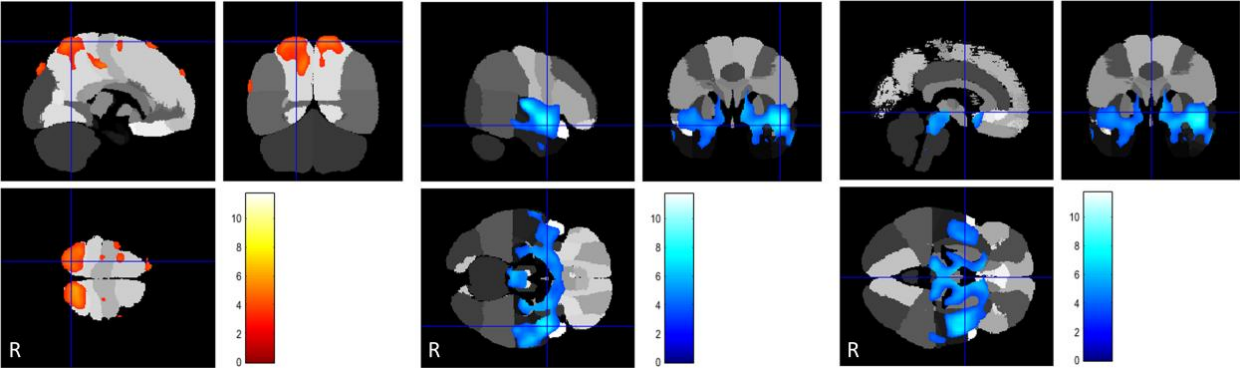
**FDG uptake in CP-preterm cases (*n* = 35) compared to controls**. *Left*: Relative hypermetabolism in the superior parietal lobes displayed in a yellow-scale with brighter tones indicating higher *t*-scores. *Middle* and *right*: Areas of relative hypometabolism, involving the superior-medial temporal lobes, peri-insular cortex, globi pallidi, thalami and brainstem, displayed in a blue-scale with brighter tones indicating higher *t*-scores.

**Figure 6:**
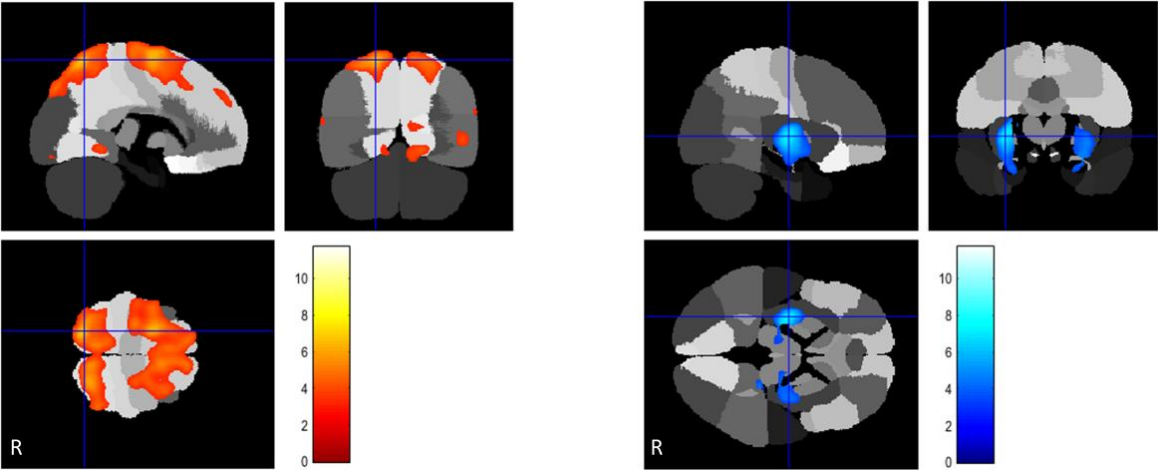
**FDG uptake in CP-term cases (*n* = 45) compared to controls**. *Left*: Regionally increased uptake in the superior anterior frontal and superior parietal lobules respectively, displayed in a yellow-scale with brighter tones indicating higher *t*-scores. *Right*: Regionally reduced uptake in the posterior putamina, globi pallidi and lateral parts of the thalami displayed in a blue-scale with brighter tones indicating higher *t*-scores.

**Table 2:** Regional FDG-PET uptake according to childhood dystonia aetiologies

Dystonia subgroup	Brain region								
	Frontal cortex	Temporal cortex	Parietal cortex	Insula	Putamen	Globus pallidus	Thalamus	Brainstem	Cerebellum
TOR1A (*n* = 7)	–	–	–	–	(−) *t* = 5.1	–	–	–	–
THAP1 (*n* = 3)	–	–	–	–	(−) *t* = 8.1	(−) *t* = 6.1	–	–	–
SGCE (*n* = 6)	(−) *t* = 5.1	–	–	–	(−) *t* = 6.5	(−) *t* = 5.8	–	–	–
KMT2B (*n* = 7)	–	(−) *t* = 5.7	–	–	(−) *t* = 8.0	(−) *t* = 7.6	–	–	–
HPRT1 (*n* = 5)	(−)	(−)	(−)	(−)	(−) *t* = 7.3	(−) *t* = 6.8	(−)	(−)	(−)
GCDH (*n* = 9)	–	–	–	–	(−) *t* = 11.7	(−) *t* = 8.8	–	–	–
PANK2 (*n* = 14)	(−) *t* = 6.7	–	(+) *t* = 6.9	(−) *t* = 4.7	–	–	–	–	(−) *t* = 5.0
CP-Kernicterus (*n* = 13)	–	(−) *t* = 6.7	(+) *t* = 5.8	(−)	–	(−)	–	(−)	(−)
CP-Preterm (*n* = 35)	–	–	(+) *t* = 6.1	(−) *t* = 8.7	–	(−)	(−)	(−)	(−)
CP-Term (*n* = 45)	(+) *t* = 6.3	–	(+) *t* = 5.7	–	(−) *t* = 7.2	(−) *t* = 4.4	(−) *t* = 4.2	–	–

The ‘+’ and ‘−’ symbols indicate the direction of statistically significant differences in regional FDG uptake between each dystonia aetiologic subgroup (rows) and the healthy controls. Maximum *t*-scores are provided. The *t*-score measures the size of the difference relative to variation in each group comparison. It is equivalent to the number of standard deviations away from the mean of the *t*-distribution. When no individual regional *t*-scores are provided, SPM clusters were confluent. (+) indicates regional hypermetabolism of the patients compared to controls, whereas (−) represents regional hypometabolism. The blank cells indicate no statistically significant difference between the patient subgroups and the controls in that particular brain region.

### Inherited forms of dystonia relative to controls

The *TOR1A* dystonia group (*n* = 7) showed relative hypometabolism in the posterior putamina. (Fig. 2A and Supplementary Fig. 1). The *THAP1* group (*n* = 3) showed relative brain hypometabolism in both the posterior putamina and the globi pallidi. (Fig. 2B and Supplementary Fig. 2). The *SGCE* group (*n* = 6) had relative hypometabolism in the ‘anterior’ putamina and ‘anterior’ globi pallidi but also in regions of the frontal cortex (Fig. 2C and Supplementary Fig. 3). In the *KMT2B* group (*n* = 7), regional relative hypometabolism was observed in the posterior putamina, globi pallidi and head of caudate bilaterally as well as some areas of the temporal cortex (Fig. 2D and Supplementary Fig. 4). *HPRT1* (*n* = 5) showed the most widespread hypometabolism involving all nine predefined areas of the brain, including the frontal, temporal, parietal and insular cortex, putamen, pallidum, thalamus, brainstem and cerebellum (Fig. 2E and Supplementary Fig. 5). The scans of patients with *GCDH* (glutaric aciduria type 1, GA-1, *n* = 9) demonstrated relative regional hypometabolism in the posterior putamina and globi pallidi (Fig. 2F and Supplementary Fig. 6). Finally, the *PANK2* group (*n* = 14) exhibited normal glucose uptake in the caudate, putamen and globi pallidi, respectively, but reduced FDG-PET-CT wglucose uptake in the peri-insular cortex, the dentate nuclei of the cerebellum and postero-inferior cerebellar cortex with hypermetabolism in the superior parietal cortex. (Fig. 3 and Supplementary Fig. 7A and B).

### Acquired forms of dystonia relative to controls

All CP cases, regardless of aetiology, were hypermetabolic in the parietal cortex relative to controls. For CP-kernicterus (*n* = 13) areas of FDG-PET-CT hypometabolism were observed in the antero-medial temporal cortex, peri-insular cortex, globi pallidi, thalami, midbrain, pons and cerebellum. Relative hypermetabolism was seen in the superior parietal lobes (Fig. 4 and Supplementary Fig. 8A and B). CP-preterm (*n* = 35) FDG-PET-CT scans showed relative hypometabolism involving the peri-insular cortex, globi pallidi, thalami, brainstem and cerebellum. Relative hypermetabolism was seen in the superior parietal lobes (Fig. 5 and Supplementary Fig. 9A and B). CP-term cases (*n* = 45) were all attributed to perinatal hypoxic-ischaemic encephalopathy (HIE). FDG-PET scans in this subgroup demonstrated a unique finding of frontal cortex hypermetabolism together with regionally reduced glucose uptake in the posterior putamina, globi pallidi and pulvinar of the thalami (Supplementary Fig. 10A and B).

### PET glucose hypometabolism by brain region

Frontal cortex hypometabolism was only found in *SGCE* (minimally affected), *HPRT1* and *PANK2* cases. Temporal cortex hypometabolism was found in *KMT2B*, *HPRT1* and CP-Kernicterus cases, while parietal cortex hypometabolism was uniquely found in the *HPRT1* cases. Insula hypometabolism was observed in *HPRT1*, *PANK2*, CP-Kernicterus and CP-Preterm. Putaminal hypometabolism occurred in 7/10 aetiological categories, except *PANK2*, CP-Kernicterus and CP-Pre-term cases. Pallidal hypometabolism was found in 8/10 diagnostic groups, except *TOR1A* and *PANK2* cases. Thalamic hypometabolism was seen in 3/10 groups, including *HPRT1*, CP-Preterm and CP-Term cases. Brainstem hypometabolism occurred in 3/10 groups, namely *HPRT1*, CP-Kernicterus and CP-Preterm. Cerebellar hypometabolism occurred in *HPRT1*, *PANK2*, CP-Kernicterus and CP-Preterm. Combined putaminal and pallidal hypometabolism occurred in 6/10 diagnostic categories, including *THAP1*, *SGCE*, *KMT2B*, *HPRT1*, *GCDH* and CP-Term, while combined cerebellar and pallidal hypometabolism was only seen in 3/10 diagnoses namely *HPRT1*, CP-kernicterus and CP-Preterm.

### PET hypermetabolism by brain region

Parietal cortex glucose hypermetabolism was found in 4/10 diagnoses, namely the three CP subgroups, CP-Kernicterus, CP-Preterm and CP-Term, and uniquely in *PANK2* cases among the genetic forms. Frontal hypermetabolism was found uniquely in CP-Term cases, which represented 45/144 (31%) cases in this study.

## Discussion

We present novel FDG-PET data demonstrating awake resting cerebral glucose metabolism in 144 children and young people with generalized dystonia, reporting comparisons of regional brain glucose metabolism across 10 distinct aetiological groups compared with 39 controls. To our knowledge, this is the largest reported cohort of FDG-PET findings in individuals with dystonia, regardless of age, and the first report of FDG-PET findings in patients with dystonia due to *KMT2B*, *HPRT1*, *GCDH*/GA-1 or in acquired dystonia/dystonic CP. The analysis revealed abnormalities that were common across aetiological groups, but also distinct differences in FDG-PET glucose metabolism distribution, which constitute a unique ‘signature’ pattern for each group. We further explored some of the aetiology-specific glucose metabolic patterns observed in our analysis and how these might relate to distinct clinical manifestations in these conditions.

### Glucose metabolic signatures of different aetiologic subgroups

We found that some brain glucose metabolism patterns were common across groups. Putaminal and pallidal hypometabolism were present, alone or in combination, in all groups except *PANK2*, pointing towards common underlying primary and secondary pathophysiological mechanisms in dystonia. Parietal hypermetabolism was found in acquired dystonias and in *PANK2*. An identical pattern of combined putaminal and pallidal hypometabolism was seen in two genetic subcategories (*THAP1* and *GCDH*/GA-1); these conditions manifest with dystonia distinguishable on clinical grounds but are often treatment-resistant.

Despite these similarities, brain glucose uptake patterns were distinct amongst different genetic dystonia subgroups. Our study revealed resting, awake putaminal glucose hypometabolism in the *TOR1A* and putamino-pallidal glucose hypotmetabolism in *THAP1*. This contrasts with previous studies which reported sensorimotor network hyperactivity in *TOR1A* dystonia^28,36^ when performing a specific motor task, and temporal cortex hypermetabolism in *THAP1.*^37^ This apparent contradiction between imaging findings could be explained by the exact conditions under which the scans were performed in the different studies (resting state versus active motor execution). Interestingly, the study showing temporal hypermetabolism^37^ also demonstrated regional putaminal hypometabolism in *THAP1* patients, concordant with our findings.

The findings of relative glucose hypometabolism in the ‘anterior’ putamina and ‘anterior’ globi pallidi and cortical areas of the frontal lobe in *SGCE* are consistent with the well-known limbic-related obsessive-compulsive disorder^29^ and less fixed dystonia co-existing with the epsilon-sarcoglycan mutation in this group. Carbon *et al*.^29^ also reported frontal lobe hypometabolism in patients with *SGCE* and additionally metabolic increases in the inferior pons and the posterior thalamus: patterns not present in our cohort.

*KMT2B* patients exhibited regional hypometabolism in the posterior putamina, globi pallidi and the head of caudate bilaterally, consistent with clinically-observed dystonia-parkinsonism features, while the areas of temporal cortex hypometabolism are consistent with the characteristic whispering dysphonia-dysarthria.^38^

The *PANK2* FDG-PET characteristics were unique, revealing bilateral glucose hypometabolism in frontal cortex, insula, dentate nuclei of the cerebellum and postero-inferior cerebellar cortex and, strikingly, bilateral superior parietal lobule hypermetabolism. The latter is not seen in *TOR1A*, *THAP1*, *SGCE*, *KMT2B* or *HPRT1* but is present in all three categories of CP-dystonia.

Surprisingly, the MRI bilateral pallidal ‘eye of the tiger’ high T_2_ signal and susceptibility-weighted feature due to increased iron deposition, which is considered a pathognomonic feature of *PANK2*,^39^ was not associated with altered pallidal metabolism compared to controls. In a previous study comparing *PANK2* with 16 cases of primary dystonia, FDG-PET revealed relative overactivity in the pallidum and underactivity in the cerebellum in *PANK2* compared to primary dystonia.^30^ In that study, the primary dystonia group comprised two *TOR1A*, two *SGCE* and twelve idiopathic cases. Therefore, the scans of *PANK2* patients were compared to a heterogeneous patient group, as opposed to a healthy control group. This methodological difference could—at least in part—be responsible for the difference in observations. Additionally, stereotactic pallidal microelectrode recordings during DBS surgery revealed distinctive patterns of basal ganglia neuronal activity in children with *PANK2*, showing predominantly regular and higher neuronal firing rates (without bursting patterns) in the globus pallidus internus compared with isolated genetic dystonias (*TOR1A* and *SGCE*) and dystonic CP (in whom relatively lower firing rates and irregular and bursting patterns of neuronal activity were seen).^40,41^ We therefore postulate that the preserved pallidal glucose metabolism in *PANK2* might reflect the presence of underlying higher pallidal neurone activity in this group compared with that in monogenic dystonias and dystonic CP. The neuronal firing patterns observed in *PANK2*, which are more similar to those seen in Parkinson’s disease, are in turn in keeping with the clinical phenotype.^41,42^ However, since the basal ganglia, thalamic and brainstem resting glucose metabolism in *PANK2* is similar to controls (Table 2), the pathological focus for an anatomical ‘source’ and ‘mechanism’ of dystonia in *PANK2* could plausibly fall to the bilateral cerebellar dentate nucleus hypometabolism. One possible interpretation is that this reflects bilateral ‘loss’ of cerebellar inhibitory control of the thalami and basal ganglia. Our study did not include other NBIA groups which would be interesting to include in future work, to establish whether these metabolic patterns are unique to *PANK2* mutations or common amongst the brain iron accumulation disorders.

It is also notable that three other distinct aetiological groups apart from *PANK2* exhibit awake resting FDG-PET cerebellar glucose hypometabolism, namely *HPRT1*, CP-Kernicterus and CP-Preterm. More research is needed to study whether this metabolism pattern is linked to a common downstream cell-based and/or neuronal network dysfunction clinically manifesting as dystonia.

Finally, standing out in contrast to all other aetiological categories was the CP-Term (HIE) group, showing significant frontal and parietal lobe hypermetabolism with hypometabolism of putamina, globi pallidi and thalami, and normal metabolism of brainstem and cerebellum. This might indicate that reduction/elimination of basal ganglia and thalamic inputs but preservation of cerebellar input results in frontal lobe hypermetabolism/disinhibition.

### Possible functional implications of regional FDG uptake patterns

Mitochondria, the organelles ultimately metabolizing glucose, are concentrated at synapses.^27^ FDG uptake is linked to synaptic density as well as activity,^25^ with 80–90% of glucose uptake in rats linked to glutamatergic (i.e. excitatory) neurotransmission.^26^ However, inhibitory synaptic activity also leads to increased glucose utilization.^43,44^ It is therefore not possible to directly interpret FDG uptake differences as reflecting specific changes in specific pathways or neurotransmitter systems.

In reviewing the FDG-PET glucose metabolic patterns, it must also be acknowledged that these are likely to reflect a combination of changes relating to the underlying genetic or acquired aetiologies and changes which are adaptive secondary consequences of long-standing dystonia, which it is outside the scope of this study to unravel. It is difficult to confidently distinguish which metabolic changes are the cause and which are the effect of dystonic symptomatology. Below, we outline our own interpretation of the glucose uptake patterns observed and their potential functional implications, within the clinical context.

Regarding cortical metabolism, only *SGCE*, *HPRT1* and *PANK2* exhibited frontal hypometabolism, which was mild in *SGCE*, and very evident in *HPRT1*, a pattern that could possibly be linked to obsessive-compulsive behaviours, which can be seen clinically in all three disorders.

*HPRT1*, *PANK2*, CP-Kernicterus and CP-Preterm all exhibited insula glucose hypometabolism. Therefore, these glucose hypometabolic patterns may point to disrupted sensorimotor, olfacto-gustatory, social-emotional and cognitive functions in these conditions, reflecting the four distinct functional regions of the insula.^45^ It is notable that all groups with cerebellar hypometabolism also had insular hypometabolism.

Examining basal ganglia metabolic patterns, putaminal hypometabolism was the sole finding in *TOR1A* and was also identified in six other aetiologic subgroups in combination with other regional changes (Table 2). In contrast, CP-Kernicterus and CP-Preterm groups showed normal putaminal glucose metabolism associated with pallidal glucose hypometabolism, whilst the *PANK2* group showed apparently normal putaminal and pallidal glucose metabolism. The unique pattern seen in *TOR1A* dystonia could plausibly be linked to the well-known responsiveness to pallidal DBS in this group.^46^ Putaminal hypometabolism could lead to altered cortical inputs and disrupted direct and indirect pathways, thus promoting dystonia. Selection of routine and reinforcement learning is disrupted by dysfunction of the putamen.^47^ Genetic dystonias may be associated with structural putaminal changes as reported in SGCE,^48^ though variable results have been described in TOR1A dystonia.^49,50^ An extreme example of early disruption of ‘routine and reinforcement learning’ is the legacy of perinatal HIE and metabolic lesions of the putamen, as seen in GCDH/GA-1.

Pallidal hypometabolism was found in 8/10 diagnoses (except *TOR1A* and *PANK2*). Loss of resting pallidal activity is potentially consistent with reduced thalamic inhibition leading to release of dystonia.

Combined putaminal and pallidal hypometabolism could be expected to produce severe dystonia via a variety of mechanisms including decreased cortical inputs to the striatum, decreased activity of medium spiny neurons or cholinergic interneurons (as well as GABA overactivity), changes in the expression of dopaminergic receptors or a mixture of all these phenomena. Glucose FDG-PET metabolic activity may also be closely linked to cholinergic systems which have been shown to be disrupted in the brain of DYT1 patients.^49^

Additionally, thalamic hypometabolism was seen in 3/10 groups, i.e. *HPRT1*, CP-Preterm and CP-Term. We propose that this is consistent with reduced capacity for direct pathway cortical activation as well as impaired or absent thalamo-parietal connectivity and sensory processing (see below). Abnormalities of sensory pathway integrity, as measured by SEPs, have been demonstrated in 47% of children with dystonia, with the majority of abnormalities seen in the acquired dystonia group.^19^ A proportion of these children had abnormalities of the thalamus on MRI,^19^ which would be consistent with hypometabolism in this region.

Finally, cerebellar FDG-PET patterns might also have functional implications. Cerebellar hypometabolism occurred in *HPRT1*, *PANK2*, CP-Kernicterus and CP-Preterm, four groups with severe baseline dystonia based on BFMDRS-M scoring. Combined cerebellar and pallidal hypometabolism was only seen in 3/10 diagnoses, namely *HPRT1*, CP-kernicterus and CP-Preterm and may be expected to favour thalamic over-activity, resulting in dystonia. *PANK2*, CP-Kernicterus, CP-Preterm and CP-Term cases exhibited awake resting parietal FDG-PET glucose hypermetabolism with several of these groups also showing cerebellar hypometabolism, indicating a pattern of possible functional association between those two brain regions. These data add to the growing recognition of dysfunctional cerebellar-network contributions to the pathogenesis of dystonia.^51^ The link between cerebellar disorders and dystonia has been supported by a variety of animal and human anatomico-physiological studies.^52–62^ Cerebellar-somatosensory cortex connectivity abnormalities in both acquired and idiopathic cervical dystonia were also reported in a recent lesion network mapping study indicating a common network abnormality between idiopathic and acquired dystonias.^17^ Such abnormalities in network mapping are pertinent particularly in childhood, during which white matter pathways exhibit differential myelination maturation windows. Cerebellar myelination extends into the late teenage years and achieves maturity over the first 16 years of life.^51^ This prolonged myelination maturation of the cerebellum gives an opportunity for cerebello-thalamic-cortical and cerebello-basal-ganglia connections to be disrupted throughout the whole of childhood in a pattern driven either by primary cerebellar pathologies or adaptive secondary cerebellar reorganization.^47^ Burbaud and colleagues^47^ summarize how cerebellar abnormalities result in impairment of temporal processing of sensorimotor information and supervised learning.

### Suggestions for future research

Overall, our findings provide new insights into paediatric dystonia in different aetiological subgroups. More neuronal network studies, including pre-clinical models, are warranted to better elucidate the pathophysiological mechanisms leading to dystonia in genetic and acquired dystonias and also to further develop FDG-PET as a tool for longitudinal tracking of brain metabolism in dystonia, monitoring efficacy of therapeutic interventions and creating predictive outcome models. In murine models FDG-PET brain imaging has been shown to be a valuable tool in deciphering disease mechanisms and potential for assessing the *in vivo* efficacy of novel therapeutics.^63,64^ Using such imaging techniques in already established models of paediatric dystonias^65,66^ would be an obvious research avenue to pursue in future studies. Follow-up pre- and post-DBS insertion FDG-PET imaging studies in childhood dystonia cohorts might also offer insights of regional brain metabolism alterations in responder and non-responder subgroups.^67–69^

### Limitations

Our 39 controls represent young adults rather than age-matched control children. Our group previously conducted analysis of FDG-PET patterns in paediatric dystonia using a ‘pseudo-control’ group of 24 children with refractory focal epilepsy but with negative MRI and with PET considered normal.^70^ However, as those scans were obtained using transmission-attenuation correction rather than CT-based attenuation correction, a systematic error was introduced, which was mainly expressed as frontal hypermetabolism in controls. This gave the false impression of frontal hypometabolism in all our dystonic subgroups and also affected—though to a lesser extent—the findings in other brain regions. We therefore considered the young adult control group used in the current analysis to represent a more valid comparison, especially as there were only trivial correlations with age for the participants under the age of 60 years selected here (data not shown). Conversely the aetiological groupings also contained children of varying age in whom primary and secondary developmental factors must certainly play a role. We aimed to minimize the impact of this by using age as a covariate in each SPM analysis. Previous literature^71^ has shown that children older than 6 years of age display very similar patterns of brain glucose utilization as adults. In our cohort, a total of 19 children were under the age of 6 years, and these were distributed across different aetiological subgroups, so we consider it unlikely that this factor would introduce a significant bias to the between-group comparisons.

While two different scanners were used for patients over the long time period of the study, one of them corresponded to the hardware used for the control group, attenuation correction was CT-based in all, and all used the same reconstruction type. Spatial resolution was similar and small differences will have been minimized by the smoothing inherent in the SPM method (via spatial normalization and explicit Gaussian smoothing).

The potential impact of different medications on PET imaging has not been examined in this study. 81/144 patients whose scans were analysed were on at least one medication for neurological symptoms (range: 1–5 medications, mean 2). These included benzodiazepines, gabapentin, clonidine, trihexyphenidyl and baclofen. The effect these medications could have on the analysis is uncertain. However, we would expect them to—if at all—have an effect on global metabolism (as shown for example for benzodiazepines by Silva-Rodriguez *et al*.^72^), rather than influence the regional patterns of metabolism described in our study. Again, these patients were distributed across the groups, so we consider that medication-use is unlikely to have a significant effect on the between-group comparisons.

Finally, the numbers in some of the aetiological sub-groups were small (e.g. *THAP1* group *n* = 3), weakening the strength of our findings in these groups. However, it should be noted that FDG-PET is routinely used clinically for abnormality detection in individual patients (*n* = 1).

## Conclusion

In conclusion, this large quantitative analysis of FDG-PET imaging provides unique insights into the common and divergent abnormalities of brain function in children and young people with dystonia arising from a range of aetiologies. We present comprehensive data from relatively large sub-cohorts of children with genetic and acquired dystonias, which have so far been sub-optimally characterized in the scientific literature. We report different regional patterns of glucose hypo- and hypermetabolism observed across different aetiologies, often in keeping with the clinical phenotypes. Our findings suggest distinct diagnostic and pathophysiological ‘signatures’ for these disorders and provide further support for the network model of dystonia. Further studies to improve our understanding of the underlying pathophysiology of dystonia, and similarities or differences between different aetiologies, will be key to understanding the differential response to therapy, including neuromodulation with DBS, and to providing a more individualized pathway of care to improve outcomes for this treatment-resistant population.

## Supplementary Material

awac439_Supplementary_Data

## Data Availability

The data that support the findings of this study are available from the corresponding author, upon reasonable request.

## References

[1] Albanese A , Bhatia K, Bressman SB, et al Phenomenology and classification of dystonia: A consensus update. Mov Disord. 2013;28:863–873. 23649720 10.1002/mds.25475 PMC3729880

[2] Lin JP , Nardocci N. Recognizing the common origins of dystonia and the development of human movement: A manifesto of unmet needs in isolated childhood dystonias. Front Neurol. 2016;7:226. 28066314 10.3389/fneur.2016.00226 PMC5165260

[3] Lumsden DE , Gimeno H, Lin JP. Classification of dystonia in childhood. Parkinsonism Relat Disord. 2016;33:138–141. 27727009 10.1016/j.parkreldis.2016.10.001

[4] Lin JP , Lumsden DE, Gimeno H, et al The impact and prognosis for dystonia in childhood including dystonic cerebral palsy: a clinical and demographic tertiary cohort study. J Neurol Neurosurg Psychiatry. 2014;85:1239–1244. 24591458 10.1136/jnnp-2013-307041

[5] Lumsden DE , Gimeno H, Elze M, et al Progression to musculoskeletal deformity in childhood dystonia. Eur J Paediatr Neurol. 2016;20:339–345. 26943984 10.1016/j.ejpn.2016.02.006

[6] Lumsden DE , Gimeno H, Tustin K, et al Interventional studies in childhood dystonia do not address the concerns of children and their carers. Eur J Paediatr Neurol. 2015;19:327–336. 25661063 10.1016/j.ejpn.2015.01.003

[7] Koy A , Lin JP, Sanger TD, et al Advances in management of movement disorders in children. Lancet Neurol. 2016;15:719–735. 27302239 10.1016/S1474-4422(16)00132-0

[8] Fehlings D , Brown L, Harvey A, et al Pharmacological and neurosurgical interventions for managing dystonia in cerebral palsy: A systematic review. Dev Med Child Neurol. 2018;60:356–366. 29405267 10.1111/dmcn.13652

[9] Shah SA , Brown P, Gimeno H, et al Application of machine learning using decision trees for prognosis of deep brain stimulation of globus Pallidus internus for children with dystonia. Front Neurol. 2020;11:825. 32849251 10.3389/fneur.2020.00825 PMC7115974

[10] Neychev VK , Gross RE, Lehericy S, et al The functional neuroanatomy of dystonia. Neurobiol Dis. 2011;42:185–201. 21303695 10.1016/j.nbd.2011.01.026 PMC3478782

[11] Hallett M . Neurophysiology of dystonia: The role of inhibition. Neurobiol Dis. 2011;42:177–184. 20817092 10.1016/j.nbd.2010.08.025 PMC3016461

[12] Quartarone A , Morgante F, Sant'angelo A, et al Abnormal plasticity of sensorimotor circuits extends beyond the affected body part in focal dystonia. J Neurol Neurosurg Psychiatr. 2008;79:985–990. 10.1136/jnnp.2007.121632 17634214

[13] Tinazzi M , Rosso T, Fiaschi A. Role of the somatosensory system in primary dystonia. Mov Disord. 2003;18:605–622. 12784263 10.1002/mds.10398

[14] McClelland VM , Cvetkovic Z, Lin JP, et al Abnormal patterns of corticomuscular and intermuscular coherence in childhood dystonia. Clin Neurophysiol. 2020;131:967–977. 32067914 10.1016/j.clinph.2020.01.012 PMC7083222

[15] Dresel C , Li Y, Wilzeck V, et al Multiple changes of functional connectivity between sensorimotor areas in focal hand dystonia. J Neurol Neurosurg Psychiatry. 2014;85:1245–1252. 24706945 10.1136/jnnp-2013-307127

[16] Rothkirch I , Granert O, Knutzen A, et al Dynamic causal modeling revealed dysfunctional effective connectivity in both, the cortico-basal-ganglia and the cerebello-cortical motor network in writers’ cramp. Neuroimage Clin. 2018;18:149–159. 29868443 10.1016/j.nicl.2018.01.015 PMC5984595

[17] Corp DT , Joutsa J, Darby RR, et al Network localization of cervical dystonia based on causal brain lesions. Brain. 2019;142:1660–1674. 31099831 10.1093/brain/awz112 PMC6536848

[18] Neumann WJ , Jha A, Bock A, et al Cortico-pallidal oscillatory connectivity in patients with dystonia. Brain. 2015;138(Pt 7):1894–1906. 25935723 10.1093/brain/awv109

[19] McClelland VM , Fialho D, Flexney-Briscoe D, et al Somatosensory evoked potentials and central motor conduction times in children with dystonia and their correlation with outcomes from deep brain stimulation of the globus pallidus internus. Clin Neurophysiol. 2018;129:473–486. 29254860 10.1016/j.clinph.2017.11.017 PMC5786451

[20] Sakellariou DF , Dall'Orso S, Burdet E, et al Abnormal microscale neuronal connectivity triggered by a proprioceptive stimulus in dystonia. Sci Rep. 2020;10:20758. 33247213 10.1038/s41598-020-77533-w PMC7695825

[21] McClelland VM , Fischer P, Foddai E, et al EEG Measures of sensorimotor processing and their development are abnormal in children with isolated dystonia and dystonic cerebral palsy. Neuroimage Clin. 2021;30: 102569. 33583764 10.1016/j.nicl.2021.102569 PMC8044718

[22] Nelson AJ , Blake DT, Chen R. Digit-specific aberrations in the primary somatosensory cortex in writer's cramp. Ann Neurol. 2009;66:146–154. 19743446 10.1002/ana.21626

[23] Fish DR , Sawyers D, Allen PJ, et al The effect of sleep on the dyskinetic movements of Parkinson's disease, gilles de la tourette syndrome, huntington's disease, and torsion dystonia. Arch Neurol. 1991;48:210–214. 1825167 10.1001/archneur.1991.00530140106023

[24] Burke RE , Fahn S, Marsden CD, et al Validity and reliability of a rating scale for the primary torsion dystonias. Neurology. 1985;35:73–77. 3966004 10.1212/wnl.35.1.73

[25] Rocher AB , Chapon F, Blaizot X, et al Resting-state brain glucose utilization as measured by PET is directly related to regional synaptophysin levels: A study in baboons. Neuroimage. 2003;20:1894–1898. 14642499 10.1016/j.neuroimage.2003.07.002

[26] Sibson NR , Dhankhar A, Mason GF, et al Stoichiometric coupling of brain glucose metabolism and glutamatergic neuronal activity. Proc Natl Acad Sci U S A. 1998;95:316–321. 9419373 10.1073/pnas.95.1.316 PMC18211

[27] Palay SL . Synapses in the central nervous system. J Biophys Biochem Cytol. 1956;2(4 Suppl):193–202. 13357542 10.1083/jcb.2.4.193 PMC2229686

[28] Carbon M , Argyelan M, Habeck C, et al Increased sensorimotor network activity in DYT1 dystonia: A functional imaging study. Brain: J Neurol. 2010;133(Pt 3):690–700. 10.1093/brain/awq017 PMC2842516 20207699

[29] Carbon M , Raymond D, Ozelius L, et al Metabolic changes in DYT11 myoclonus-dystonia. Neurology. 2013;80:385–391. 23284065 10.1212/WNL.0b013e31827f0798 PMC3589244

[30] Szyszko TA , Dunn JT, O’Doherty MJ, et al Role of 18 F FDG PET imaging in paediatric primary dystonia and dystonia arising from neurodegeneration with brain iron accumulation. Nucl Med Commun. 2015;36:469–476. 25646707 10.1097/MNM.0000000000000273

[31] Guedj E , Varrone A, Boellaard R, et al EANM Procedure guidelines for brain PET imaging using [(18)F]FDG, version 3. Eur J Nucl Med Mol Imaging. 2022;49:632–651. 34882261 10.1007/s00259-021-05603-w PMC8803744

[32] Ashburner J , Friston KJ. Unified segmentation. Neuroimage. 2005;26:839–851. 15955494 10.1016/j.neuroimage.2005.02.018

[33] Hammers A , Allom R, Koepp MJ, et al Three-dimensional maximum probability atlas of the human brain, with particular reference to the temporal lobe. Hum Brain Mapp. 2003;19:224–247. 12874777 10.1002/hbm.10123 PMC6871794

[34] Hammers A , Chen CH, Lemieux L, et al Statistical neuroanatomy of the human inferior frontal gyrus and probabilistic atlas in a standard stereotaxic space. Hum Brain Mapp. 2007;28:34–48. 16671082 10.1002/hbm.20254 PMC6871382

[35] Ahsan RL , Allom R, Gousias IS, et al Volumes, spatial extents and a probabilistic atlas of the human basal ganglia and thalamus. Neuroimage. 2007;38:261–270. 17851093 10.1016/j.neuroimage.2007.06.004

[36] Niethammer M , Carbon M, Argyelan M, et al Hereditary dystonia as a neurodevelopmental circuit disorder: Evidence from neuroimaging. Neurobiol Dis. 2011;42:202–209. 20965251 10.1016/j.nbd.2010.10.010 PMC3062649

[37] Carbon M , Su S, Dhawan V, et al Regional metabolism in primary torsion dystonia: Effects of penetrance and genotype. Neurology. 2004;62:1384–1390. 15111678 10.1212/01.wnl.0000120541.97467.fe

[38] Cif L , Demailly D, Lin JP, et al KMT2B-related Disorders: expansion of the phenotypic spectrum and long-term efficacy of deep brain stimulation. Brain. 2020;143:3242–3261. 33150406 10.1093/brain/awaa304 PMC7719027

[39] Renaud DL , Kotagal S. Pantothenate-Kinase Associated Neurodegeneration (PKAN) “eye of the tiger” sign. Pediatr Neurol. 2007;36:70–71. 17162204 10.1016/j.pediatrneurol.2006.09.005

[40] McClelland VM , Valentin A, Rey HG, et al Differences in globus pallidus neuronal firing rates and patterns relate to different disease biology in children with dystonia. J Neurol Neurosurg Psychiatry. 2016;87:958–967. 26848170 10.1136/jnnp-2015-311803 PMC5013118

[41] McClelland VM , Lumsden DE, Lin JP. Disease-specific patterns of basal ganglia neuronal activity in Neurodegeneration with Brain Iron Accumulation type I (NBIA-1). Clin Neurophysiol. 2019;130:877–878. 30978626 10.1016/j.clinph.2019.03.005

[42] Huebl J , Poshtiban A, Brücke C, et al Subthalamic and pallidal oscillatory activity in patients with Neurodegeneration with Brain Iron Accumulation type I (NBIA-I). Clin Neurophysiol. 2019;130:469–473. 30771723 10.1016/j.clinph.2018.12.012

[43] Nudo RJ , Masterton RB. Stimulation-induced [14C]2-deoxyglucose labeling of synaptic activity in the central auditory system. J Comp Neurol. 1986;245:553–565. 3009561 10.1002/cne.902450410

[44] Ackermann RF , Finch DM, Babb TL, et al Increased glucose metabolism during long-duration recurrent inhibition of hippocampal pyramidal cells. J Neurosci. 1984;4:251–264. 6693941 10.1523/JNEUROSCI.04-01-00251.1984 PMC6564752

[45] Kurth F , Zilles K, Fox PT, et al A link between the systems: functional differentiation and integration within the human insula revealed by meta-analysis. Brain Struct Funct. 2010;214(5–6):519–534. 20512376 10.1007/s00429-010-0255-z PMC4801482

[46] Moro E , LeReun C, Krauss JK, et al Efficacy of pallidal stimulation in isolated dystonia: A systematic review and meta-analysis. Eur J Neurol. 2017;24:552–560. 28186378 10.1111/ene.13255 PMC5763380

[47] Burbaud P , Courtin E, Ribot B, et al Basal ganglia: From the bench to the bed. Eur J Paediatr Neurol. 2022;36:99–106. 34953339 10.1016/j.ejpn.2021.12.002

[48] Beukers RJ , van der Meer JN, van der Salm SM, et al Severity of dystonia is correlated with putaminal gray matter changes in myoclonus-dystonia. Eur J Neurol. 2011;18:906–912. 21219543 10.1111/j.1468-1331.2010.03321.x

[49] Mazere J , Dilharreguy B, Catheline G, et al Erratum to: Striatal and cerebellar vesicular acetylcholine transporter expression is disrupted in human DYT1 dystonia. Brain. 2021;144:e68. 34323950 10.1093/brain/awab172

[50] Draganski B , Schneider SA, Fiorio M, et al Genotype-phenotype interactions in primary dystonias revealed by differential changes in brain structure. Neuroimage. 2009;47:1141–1147. 19344776 10.1016/j.neuroimage.2009.03.057 PMC2741581

[51] Sival DA , Noort S, Tijssen MAJ, et al Developmental neurobiology of cerebellar and basal ganglia connections. Eur J Paediatr Neurol. 2022;36:123–129. 34954622 10.1016/j.ejpn.2021.12.001

[52] Raike RS , Hess EJ, Jinnah HA. Dystonia and cerebellar degeneration in the leaner mouse mutant. Brain Res. 2015;1611:56–64. 25791619 10.1016/j.brainres.2015.03.011 PMC4441875

[53] Ramnani N . The primate cortico-cerebellar system: anatomy and function. Nat Rev Neurosci. 2006;7:511–522. 16791141 10.1038/nrn1953

[54] Milardi D , Quartarone A, Bramanti A, et al The cortico-basal ganglia-cerebellar network: Past, present and future perspectives. Front Syst Neurosci. 2019;13:61. 31736719 10.3389/fnsys.2019.00061 PMC6831548

[55] Middleton FA , Strick PL. Basal ganglia and cerebellar loops: Motor and cognitive circuits. Brain Res Brain Res Rev. 2000;31(2–3):236–250. 10719151 10.1016/s0165-0173(99)00040-5

[56] Hubsch C , Roze E, Popa T, et al Defective cerebellar control of cortical plasticity in writer's cramp. Brain 2013; 136(Pt ):2050–2062. 23801734 10.1093/brain/awt147 PMC3692031

[57] White JJ , Sillitoe RV. Genetic silencing of olivocerebellar synapses causes dystonia-like behaviour in mice. Nat Commun. 2017;8:14912. 28374839 10.1038/ncomms14912 PMC5382291

[58] Dean P , Porrill J. The cerebellum as an adaptive filter: a general model? Funct Neurol. 2010;25:173–180. 21375070

[59] Le Ber I , Clot F, Vercueil L, et al Predominant dystonia with marked cerebellar atrophy: A rare phenotype in familial dystonia. Neurology. 2006;67:1769–1773. 17130408 10.1212/01.wnl.0000244484.60489.50

[60] Bologna M , Berardelli A. Cerebellum: an explanation for dystonia? Cerebellum Ataxias. 2017;4:6. 28515949 10.1186/s40673-017-0064-8 PMC5429509

[61] Batla A , Sánchez MC, Erro R, et al The role of cerebellum in patients with late onset cervical/segmental dystonia? –evidence from the clinic. Parkinsonism Relat Disord. 2015;21:1317–1322. 26385708 10.1016/j.parkreldis.2015.09.013

[62] Zoons E , Tijssen MA. Pathologic changes in the brain in cervical dystonia pre- and post-mortem—A commentary with a special focus on the cerebellum. Exp Neurol. 2013;247:130–133. 23597638 10.1016/j.expneurol.2013.04.005

[63] Bouter C , Bouter Y. (18)F-FDG-PET in mouse models of Alzheimer's disease. Front Med (Lausanne). 2019;6:71. 31058151 10.3389/fmed.2019.00071 PMC6482246

[64] Welch A , Mingarelli M, Riedel G, et al Mapping changes in mouse brain metabolism with PET/CT. J Nucl Med. 2013;54:1946–1953. 24009277 10.2967/jnumed.113.121509

[65] Subramanian C , Yao J, Frank MW, et al A pantothenate kinase-deficient mouse model reveals a gene expression program associated with brain coenzyme a reduction. Biochim Biophys Acta Mol Basis Dis 2020;1866(5):165663. 31918006 10.1016/j.bbadis.2020.165663 PMC7078592

[66] Witteveen JS , Loopstok SR, Ballesteros LL, et al HGprt deficiency disrupts dopaminergic circuit development in a genetic mouse model of lesch-nyhan disease. Cell Mol Life Sci. 2022;79:341. 35660973 10.1007/s00018-022-04326-x PMC9167210

[67] Hilker R , Voges J, Weber T, et al STN-DBS activates the target area in Parkinson disease: An FDG-PET study. Neurology. 2008;71:708–713. 18650492 10.1212/01.wnl.0000312380.01852.77

[68] Kalbe E , Voges J, Weber T, et al Frontal FDG-PET activity correlates with cognitive outcome after STN-DBS in Parkinson disease. Neurology. 2009;72:42–49. 19122029 10.1212/01.wnl.0000338536.31388.f0

[69] Peng S , Dhawan V, Eidelberg D, et al Neuroimaging evaluation of deep brain stimulation in the treatment of representative neurodegenerative and neuropsychiatric disorders. Bioelectron Med. 2021;7:4. 33781350 10.1186/s42234-021-00065-9 PMC8008578

[70] Archambaud F , Bouilleret V, Hertz-Pannier L, et al Optimizing statistical parametric mapping analysis of 18F-FDG PET in children. EJNMMI Res. 2013;3:2. 23289862 10.1186/2191-219X-3-2 PMC3558387

[71] Muzik O , Chugani DC, Juhász C, et al Statistical parametric mapping: Assessment of application in children. Neuroimage. 2000;12:538–549. 11034861 10.1006/nimg.2000.0651

[72] Silva-Rodríguez J , García-Varela L, López-Arias E, et al Impact of benzodiazepines on brain FDG-PET quantification after single-dose and chronic administration in rats. Nucl Med Biol. 2016;43:827–834. 27792990 10.1016/j.nucmedbio.2016.09.001

