# Supplementary material for: Metabolic patterns in brain 18F-fluorodeoxyglucose PET relate to aetiology in paediatric dystonia
Source: Brain. 2022 Nov 29;146(6):2512–23. doi: 10.1093/brain/awac439 (PMC10232264; doi:10.1093/brain/awac439)
Supplement: awac439_Supplementary_Data [file awac439_supplementary_data.pdf]

Supplementary file 1 (with supplementary figures 1-10) shows results of all comparisons between aetiologic subgroups of dystonia and controls as the output in SPM's graphics window. For each aetiologic sub-group, a single page of data is shown containing the following: a maximum intensity projection (MIP) on a glass brain in three orthogonal planes (top left), the design matrix (top right) and the results table (bottom). As the visualization threshold was set at $p<0.001$, all voxels that fulfil this criterion will show up in the MIP. The SPM results table organizes those in clusters in order of decreasing T-score. For our analysis a cluster of voxels was considered statistically significant if $p<0.05$, taking into consideration both effect size and spatial extent. Thus, we only reported findings that satisfied this latter criterion.
A second supplementary file combines figures 2-6 of the main text into a single poster, in order to facilitate comparisons by the reader.

Supplementary Fig. 1: TOR1A<Controls

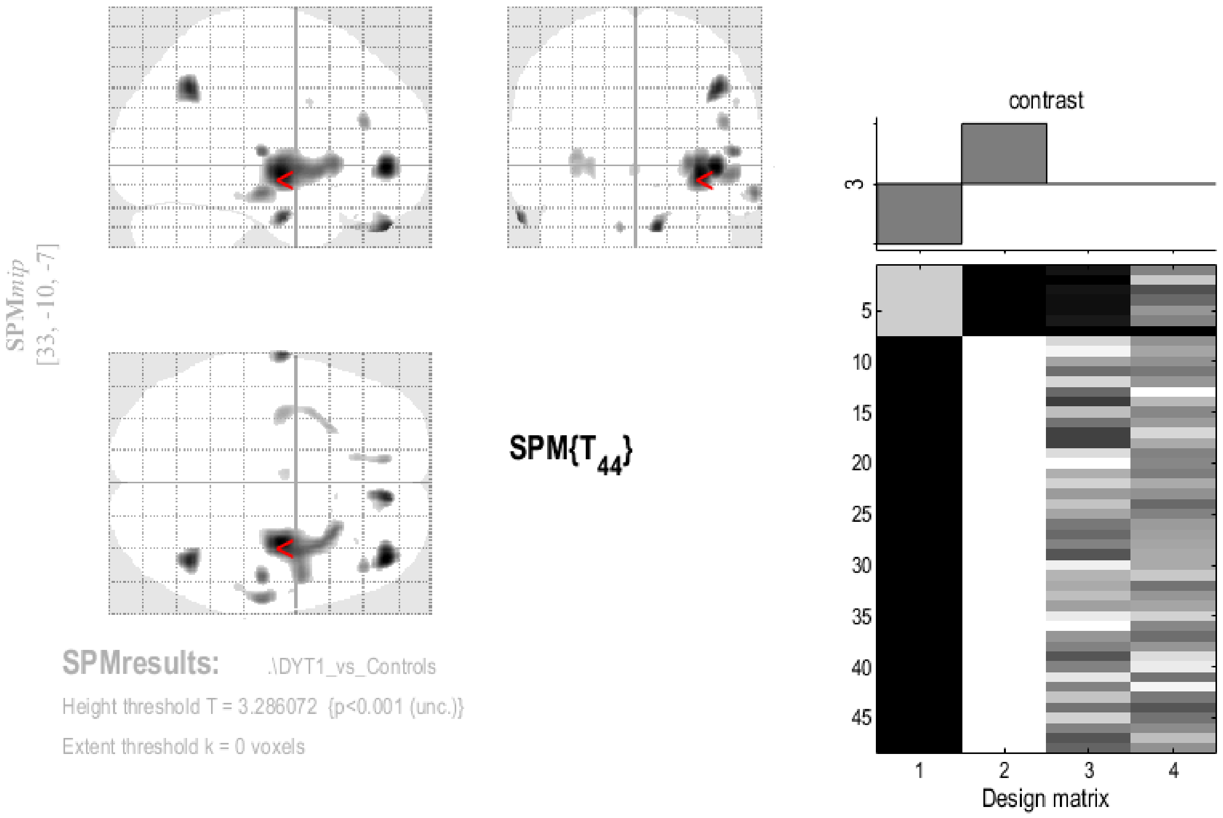

Statistics: ***p-values adjusted for search volume***

set-level		cluster-level				peak-level					mm mm mm		
p	c	$p_{FWE-corr}$	$q_{FDR-corr}$	k_E	p_{uncorr}	$p_{FWE-corr}$	$q_{FDR-corr}$	T	$(Z_{\equiv})$	p_{uncorr}			
0.000	18	0.245	0.393	1665	0.055	0.051	0.174	5.14	4.53	0.000	41	46	-3
		0.001	0.002	8984	0.000	0.064	0.174	5.06	4.47	0.000	33	-10	-7
						0.451	0.392	4.23	3.85	0.000	35	8	-5
						0.452	0.392	4.23	3.85	0.000	28	18	-2
		0.358	0.393	1290	0.087	0.123	0.182	4.81	4.29	0.000	42	-59	38
		0.686	0.680	610	0.227	0.129	0.182	4.79	4.27	0.000	10	47	-34
		0.867	0.760	302	0.395	0.211	0.251	4.59	4.12	0.000	-64	-9	-29
		0.845	0.760	342	0.364	0.403	0.392	4.29	3.90	0.000	51	-20	5
		0.608	0.659	746	0.183	0.488	0.392	4.18	3.82	0.000	62	-20	-16
						0.982	0.982	3.40	3.19	0.001	56	-33	-18
		0.877	0.760	285	0.409	0.711	0.595	3.93	3.62	0.000	45	34	21
		0.885	0.760	270	0.422	0.760	0.627	3.87	3.57	0.000	-10	45	-35
						0.950	0.982	3.54	3.30	0.000	-11	31	-34
		0.361	0.393	1283	0.087	0.850	0.772	3.75	3.47	0.000	-34	-9	2
						0.897	0.803	3.67	3.41	0.000	-34	7	-4
						0.898	0.803	3.66	3.40	0.000	-27	16	-2
		0.983	0.968	36	0.799	0.976	0.982	3.44	3.22	0.001	52	5	31
		0.950	0.958	132	0.586	0.977	0.982	3.43	3.21	0.001	-3	-8	-5
		0.988	0.968	17	0.872	0.988	0.982	3.36	3.15	0.001	57	-4	-28
		0.991	0.968	7	0.927	0.991	0.982	3.32	3.12	0.001	31	29	-29

table shows 3 local maxima more than 8.0mm apart

Height threshold: T = 3.29, p = 0.001 (0.994)
Extent threshold: k = 0 voxels
Expected voxels per cluster, <k> = 448.359
Expected number of clusters, <c> = 5.11
FWEp: 5.152, FDRp: Inf, FWEc: 8984, FDRc: 8984

Degrees of freedom = [1.0, 44.0]
FWHM = 15.7 16.4 17.3 mm mm mm; 15.7 16.4 17.3 {voxels}
Volume: 1672601 = 1672601 voxels = 355.6 resels
Voxel size: 1.0 1.0 1.0 mm mm mm; (resel = 4471.70 voxels)
Page 1

Supplementary Fig. 2: THAP1<Controls

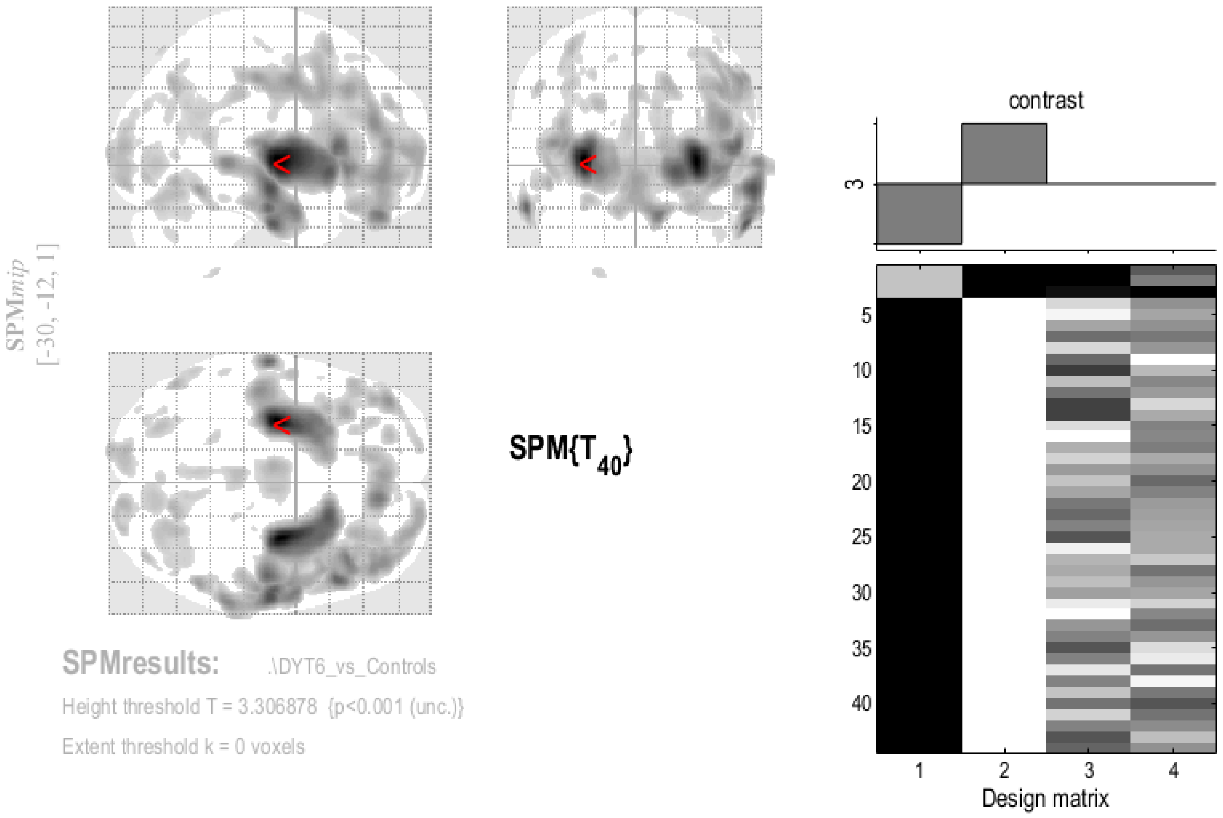

Statistics: ***p-values adjusted for search volume***

set-level		cluster-level				peak-level					mm mm mm		
p	c	$p_{FWE\text{-}corr}$	$q_{FDR\text{-}corr}$	k_E	p_{uncorr}	$p_{FWE\text{-}corr}$	$q_{FDR\text{-}corr}$	T	$(Z_{\equiv})$	p_{uncorr}			
0.000	42	0.000	0.000	33489	0.000	0.000	0.000	8.11	6.20	0.000	-30	-12	1
						0.079	0.131	5.07	4.43	0.000	-25	14	-5
						0.137	0.189	4.85	4.27	0.000	-46	-21	10
		0.000	0.000	72921	0.000	0.000	0.000	7.93	6.11	0.000	30	-10	0
						0.005	0.026	6.07	5.08	0.000	20	16	-5
						0.012	0.037	5.80	4.91	0.000	52	20	8
		0.000	0.000	11642	0.000	0.004	0.025	6.22	5.17	0.000	61	-16	-14
						0.006	0.026	6.01	5.04	0.000	57	-14	-33
						0.044	0.084	5.30	4.59	0.000	59	-3	-26
		0.246	0.220	1613	0.052	0.014	0.037	5.71	4.86	0.000	-60	-17	-24
		0.578	0.481	789	0.160	0.200	0.219	4.69	4.16	0.000	54	-54	-14
		0.004	0.007	6318	0.001	0.467	0.385	4.27	3.85	0.000	-6	-14	-12
						0.760	0.516	3.93	3.59	0.000	5	-13	-3
		0.475	0.387	985	0.120	0.476	0.385	4.26	3.85	0.000	-49	-67	-11
						0.849	0.599	3.80	3.49	0.000	-56	-56	-11
		0.102	0.105	2467	0.020	0.497	0.385	4.24	3.83	0.000	15	-68	41
						0.891	0.643	3.73	3.44	0.000	9	-60	30
						0.921	0.698	3.67	3.39	0.000	9	-56	18
		0.066	0.076	2911	0.013	0.508	0.385	4.22	3.82	0.000	13	-94	8
						0.708	0.493	3.99	3.64	0.000	16	-98	-1

table shows 3 local maxima more than 8.0mm apart

Height threshold: T = 3.31, p = 0.001 (0.995)
Extent threshold: k = 0 voxels
Expected voxels per cluster, <k> = 423.566
Expected number of clusters, <c> = 5.38
FWEp: 5.249, FDRp: 5.715, FWEc: 3567, FDRc: 6318

Degrees of freedom = [1.0, 40.0]
FWHM = 15.5 16.2 17.1 mm mm mm; 15.5 16.2 17.1 {voxels}
Volume: 1673525 = 1673525 voxels = 370.2 resels
Voxel size: 1.0 1.0 1.0 mm mm mm; (resel = 4297.72 voxels)
Page 1

< >

Supplementary Fig. 3: SGCE<Controls

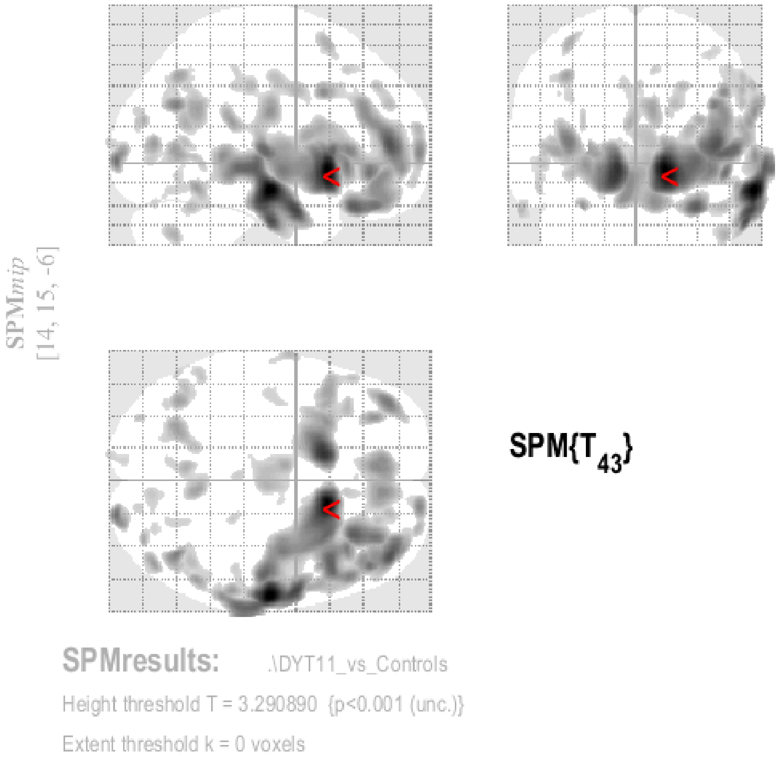

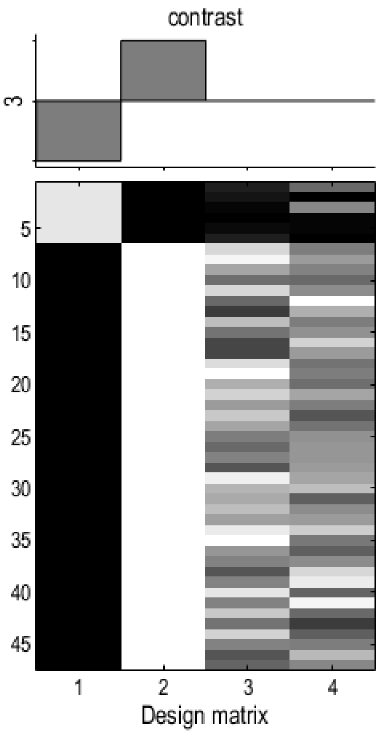

Statistics: ***p-values adjusted for search volume***

set-level		cluster-level				peak-level					mm mm mm		
p	c	$p_{FWE-corr}$	$q_{FDR-corr}$	k_E	p_{uncorr}	$p_{FWE-corr}$	$q_{FDR-corr}$	T	$(Z_{\equiv})$	p_{uncorr}			
**0.000**	**40**	**0.000**	**0.000**	**13422**	**0.000**	**0.001**	**0.012**	**6.59**	**5.45**	**0.000**	**62**	**-16**	**-16**
						0.023	0.094	5.45	4.73	0.000	59	-2	-26
						0.028	0.094	5.39	4.68	0.000	57	-15	-32
		**0.000**	**0.000**	**45348**	**0.000**	**0.001**	**0.012**	**6.48**	**5.38**	**0.000**	**14**	**15**	**-6**
						0.037	0.109	5.28	4.61	0.000	41	48	8
						0.065	0.117	5.07	4.46	0.000	48	17	4
		**0.000**	**0.000**	**18303**	**0.000**	**0.010**	**0.068**	**5.75**	**4.93**	**0.000**	**-12**	**13**	**-6**
						0.404	0.301	4.30	3.90	0.000	-29	14	-3
						0.527	0.354	4.15	3.78	0.000	-44	15	4
		**0.000**	**0.000**	**11521**	**0.000**	**0.055**	**0.117**	**5.13**	**4.51**	**0.000**	**30**	**29**	**-25**
						0.126	0.160	4.82	4.28	0.000	31	40	-24
						0.271	0.268	4.49	4.04	0.000	-5	42	-12
		**0.386**	**0.424**	**1214**	**0.095**	**0.122**	**0.160**	**4.83**	**4.29**	**0.000**	**20**	**-99**	**-1**
		**0.101**	**0.139**	**2569**	**0.021**	**0.158**	**0.194**	**4.72**	**4.22**	**0.000**	**-38**	**48**	**9**
		**0.513**	**0.510**	**927**	**0.140**	**0.195**	**0.209**	**4.64**	**4.15**	**0.000**	**40**	**-66**	**28**
		**0.181**	**0.223**	**1970**	**0.039**	**0.257**	**0.262**	**4.51**	**4.06**	**0.000**	**16**	**64**	**4**
						0.740	0.516	3.90	3.59	0.000	27	63	15
						0.933	0.753	3.59	3.34	0.000	14	61	-11
		**0.821**	**0.655**	**384**	**0.336**	**0.366**	**0.281**	**4.35**	**3.93**	**0.000**	**-31**	**40**	**-24**
		**0.673**	**0.655**	**633**	**0.218**	**0.449**	**0.336**	**4.24**	**3.85**	**0.000**	**45**	**-78**	**8**

table shows 3 local maxima more than 8.0mm apart

Height threshold: T = 3.29, p = 0.001 (0.994)
Extent threshold: k = 0 voxels
Expected voxels per cluster, <k> = 447.934
Expected number of clusters, <c> = 5.12
FWEp: 5.170, FDRp: 6.483, FWEc: 11521, FDRc: 11521

Degrees of freedom = [1.0, 43.0]
FWHM = 15.7 16.5 17.3 mm mm mm; 15.7 16.5 17.3 {voxels}
Volume: 1671903 = 1671903 voxels = 354.3 resels
Voxel size: 1.0 1.0 1.0 mm mm mm; (resel = 4485.33 voxels)
Page 1

< >

Supplementary Fig. 4: KMT2B<Controls

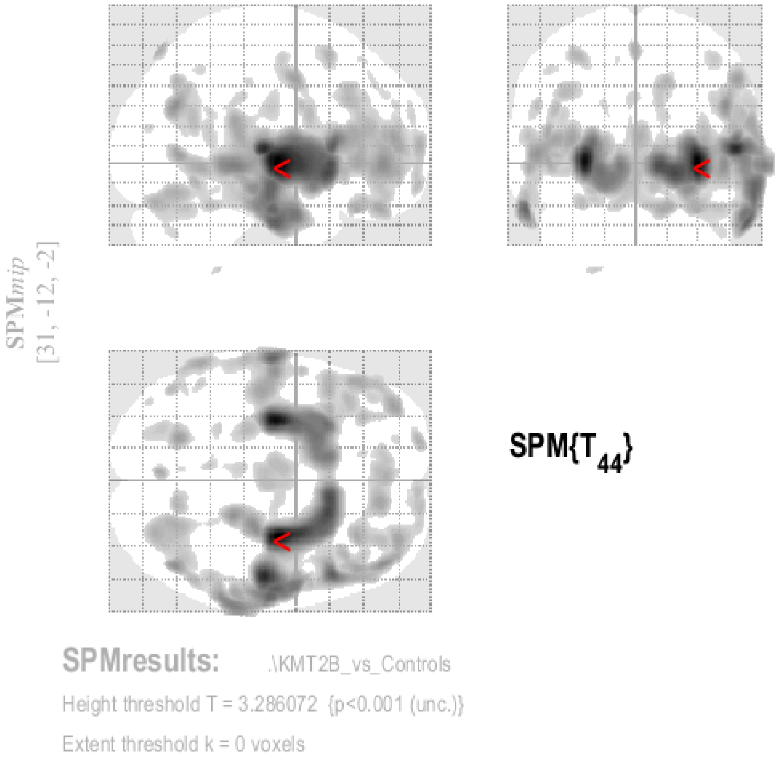

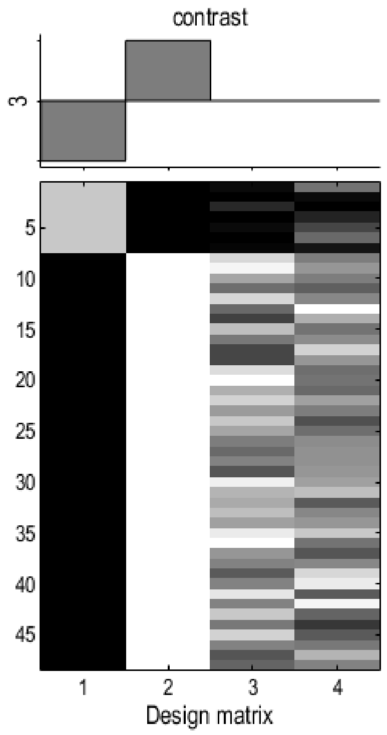

Statistics: ***p-values adjusted for search volume***

set-level		cluster-level				peak-level					mm mm mm		
p	c	$p_{FWE-corr}$	$q_{FDR-corr}$	k_E	p_{uncorr}	$p_{FWE-corr}$	$q_{FDR-corr}$	T	$(Z_{\equiv})$	p_{uncorr}			
**0.000**	**28**	**0.000**	**0.000**	**128227**	**0.000**	**0.000**	**0.000**	**7.97**	**6.24**	**0.000**	**31**	**-12**	**-2**
						0.000	0.000	7.57	6.03	0.000	-29	-13	-1
						0.000	0.002	6.92	5.66	0.000	51	-19	6
		**0.011**	**0.024**	**5161**	**0.002**	**0.009**	**0.025**	**5.74**	**4.93**	**0.000**	**-61**	**-15**	**-26**
						0.336	0.283	4.37	3.96	0.000	-59	-53	-4
						0.529	0.336	4.12	3.77	0.000	-61	-29	-19
		**0.089**	**0.131**	**2731**	**0.019**	**0.116**	**0.147**	**4.82**	**4.30**	**0.000**	**46**	**-67**	**27**
						0.612	0.369	4.03	3.70	0.000	53	-57	21
		**0.470**	**0.474**	**1017**	**0.127**	**0.201**	**0.229**	**4.60**	**4.13**	**0.000**	**-20**	**-65**	**2**
		**0.903**	**0.727**	**228**	**0.467**	**0.364**	**0.285**	**4.33**	**3.93**	**0.000**	**-2**	**-75**	**56**
		**0.013**	**0.024**	**5013**	**0.003**	**0.425**	**0.329**	**4.25**	**3.87**	**0.000**	**29**	**-77**	**-22**
						0.457	0.336	4.21	3.84	0.000	25	-66	-17
						0.798	0.504	3.81	3.52	0.000	43	-61	-26
		**0.521**	**0.474**	**910**	**0.148**	**0.434**	**0.329**	**4.24**	**3.86**	**0.000**	**-18**	**64**	**7**
		**0.533**	**0.474**	**887**	**0.152**	**0.476**	**0.336**	**4.19**	**3.82**	**0.000**	**-34**	**16**	**54**
		**0.454**	**0.474**	**1053**	**0.121**	**0.521**	**0.336**	**4.13**	**3.78**	**0.000**	**14**	**-66**	**40**
						0.832	0.536	3.76	3.48	0.000	11	-64	32
		**0.480**	**0.474**	**997**	**0.131**	**0.565**	**0.352**	**4.08**	**3.74**	**0.000**	**-39**	**26**	**35**
		**0.669**	**0.619**	**637**	**0.221**	**0.584**	**0.352**	**4.06**	**3.72**	**0.000**	**-45**	**-69**	**26**
						0.957	0.723	3.51	3.27	0.001	-47	-63	16

table shows 3 local maxima more than 8.0mm apart

Height threshold: T = 3.29, p = 0.001 (0.993)
Extent threshold: k = 0 voxels
Expected voxels per cluster, <k> = 456.908
Expected number of clusters, <c> = 4.99
FWEp: 5.143, FDRp: 5.412, FWEc: 5013, FDRc: 5013

Degrees of freedom = [1.0, 44.0]
FWHM = 15.8 16.5 17.4 mm mm mm; 15.8 16.5 17.4 {voxels}
Volume: 1654792 = 1654792 voxels = 344.9 resels
Voxel size: 1.0 1.0 1.0 mm mm mm; (resel = 4556.96 voxels)
Page 1

< >

Supplementary Fig. 5: HPRT1<Controls

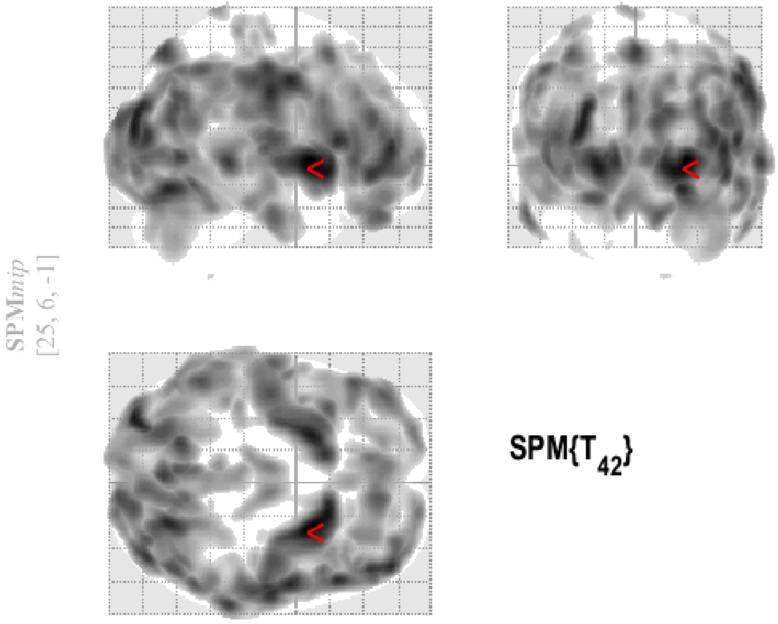

SPM{T_{42}}

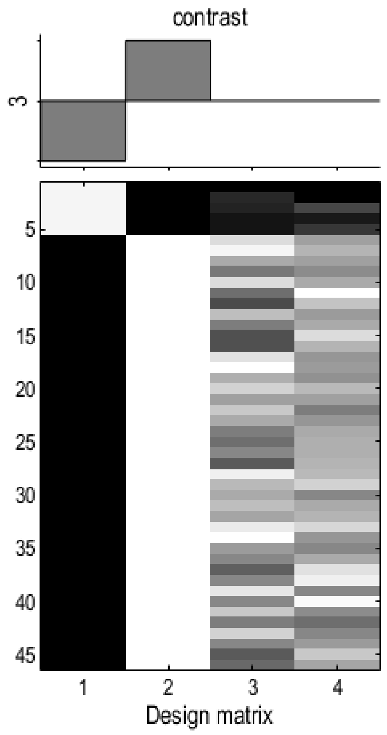

SPMresults: .\LND_vs_Controls

Height threshold T = 3.295951 {p<0.001 (unc.)}

Extent threshold k = 0 voxels

Statistics: *p-values adjusted for search volume*

set-level		cluster-level				peak-level							
p	c	$p_{FWE-corr}$	$q_{FDR-corr}$	k_E	p_{uncorr}	$p_{FWE-corr}$	$q_{FDR-corr}$	T	$(Z_{\equiv})$	p_{uncorr}	mm	mm	mm
0.156	8	0.000	0.000	434397	0.000	0.000	0.004	7.27	5.82	0.000	25	6	-1
						0.001	0.004	6.82	5.56	0.000	19	14	-5
						0.001	0.004	6.71	5.50	0.000	-22	7	-3
		0.657	0.752	655	0.205	0.237	0.084	4.57	4.09	0.000	-6	-43	79
		0.818	0.752	389	0.327	0.889	0.453	3.70	3.43	0.000	-28	34	-22
		0.913	0.752	216	0.470	0.931	0.545	3.61	3.35	0.000	29	-20	74
		0.978	0.933	56	0.736	0.936	0.552	3.60	3.34	0.000	14	-47	-59
		0.893	0.752	255	0.430	0.946	0.581	3.57	3.32	0.000	-35	-72	-43
						0.994	0.974	3.31	3.10	0.001	-27	-74	-49
		0.986	0.933	28	0.824	0.990	0.902	3.35	3.14	0.001	-16	-80	-43
		0.992	0.933	6	0.933	0.992	0.927	3.34	3.12	0.001	15	19	70

table shows 3 local maxima more than 8.0mm apart

Height threshold: T = 3.30, p = 0.001 (0.995)
Extent threshold: k = 0 voxels
Expected voxels per cluster, <k> = 437.252
Expected number of clusters, <c> = 5.21
FWEp: 5.195, FDRp: 4.917, FWEc: 434397, FDRc: 434397

Degrees of freedom = [1.0, 42.0]
FWHM = 15.6 16.3 17.2 mm mm mm; 15.6 16.3 17.2 {voxels}
Volume: 1661366 = 1661366 voxels = 359.0 resels
Voxel size: 1.0 1.0 1.0 mm mm mm; (resel = 4396.74 voxels)

Supplementary Fig. 6: GCDH<Controls

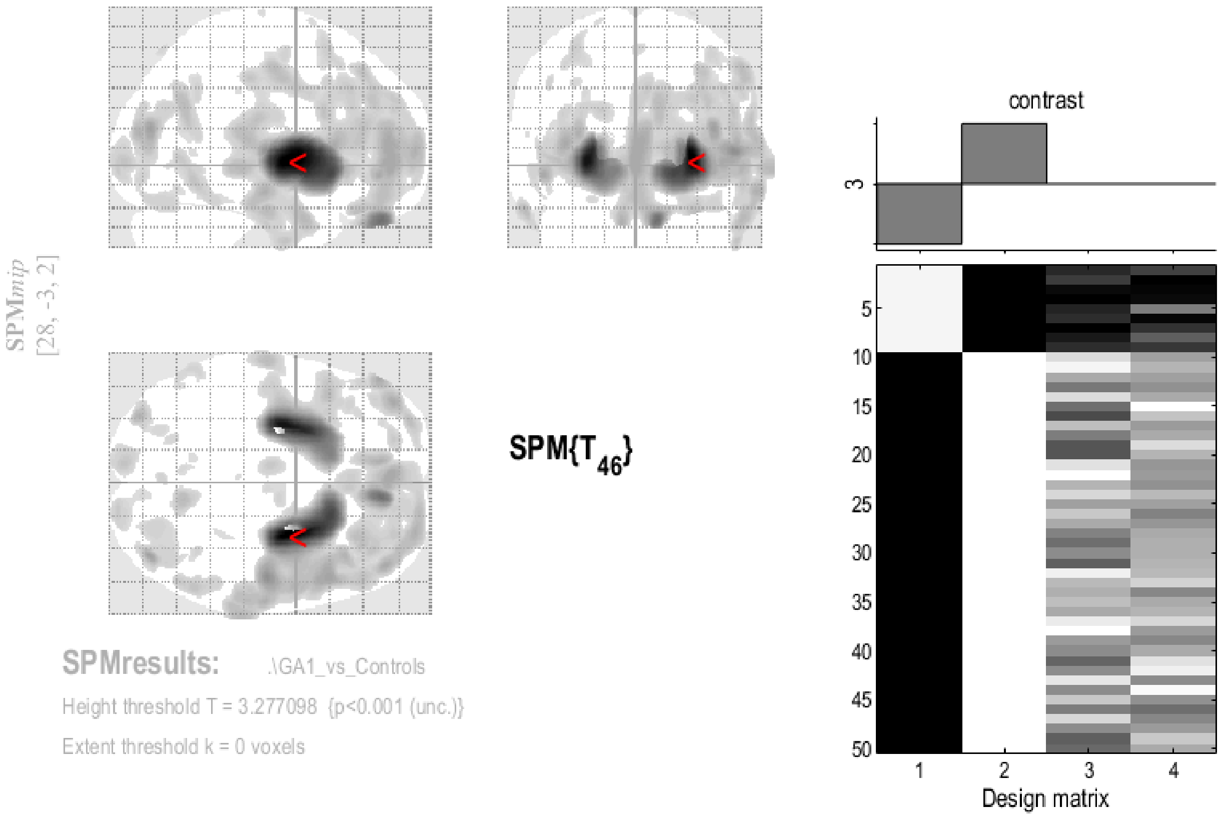

Statistics: *p-values adjusted for search volume*

set-level		cluster-level				peak-level							
p	c	$p_{FWE\text{-}corr}$	$q_{FDR\text{-}corr}$	k_E	p_{uncorr}	$p_{FWE\text{-}corr}$	$q_{FDR\text{-}corr}$	T	$(Z_{\equiv})$	p_{uncorr}	mm	mm	mm
0.000	34	0.000	0.000	94581	0.000	0.000	0.000	11.69	Inf	0.000	28	-3	2
						0.000	0.000	8.81	6.71	0.000	19	16	-6
						0.000	0.000	7.28	5.91	0.000	10	44	-30
		0.000	0.000	29264	0.000	0.000	0.000	10.78	7.57	0.000	-28	-9	0
						0.047	0.049	5.12	4.53	0.000	-51	43	-7
						0.051	0.049	5.09	4.51	0.000	-40	50	9
		0.050	0.120	3415	0.011	0.012	0.014	5.61	4.87	0.000	40	-80	17
						0.715	0.428	3.88	3.59	0.000	43	-72	27
						0.855	0.536	3.70	3.44	0.000	39	-83	-8
		0.165	0.157	2094	0.038	0.085	0.073	4.90	4.37	0.000	-33	-86	15
						0.847	0.536	3.71	3.45	0.000	-38	-85	-9
		0.343	0.259	1340	0.087	0.171	0.123	4.63	4.17	0.000	-61	-17	-26
		0.107	0.157	2554	0.024	0.183	0.126	4.60	4.15	0.000	11	-62	30
		0.199	0.157	1899	0.046	0.392	0.240	4.25	3.89	0.000	55	-57	-10
						0.550	0.318	4.07	3.74	0.000	53	-64	1
						0.753	0.460	3.84	3.55	0.000	54	-47	-20
		0.189	0.157	1954	0.044	0.425	0.251	4.21	3.85	0.000	-8	-94	0
		0.595	0.449	762	0.188	0.511	0.304	4.11	3.77	0.000	-7	-16	-15
						0.989	0.940	3.31	3.12	0.001	-4	-17	-3
		0.115	0.157	2479	0.025	0.521	0.307	4.10	3.76	0.000	17	-97	-4

table shows 3 local maxima more than 8.0mm apart

Height threshold: T = 3.28, p = 0.001 (0.992)
Extent threshold: k = 0 voxels
Expected voxels per cluster, <k> = 467.963
Expected number of clusters, <c> = 4.81
FWEp: 5.096, FDRp: 5.090, FWEc: 3415, FDRc: 29264

Degrees of freedom = [1.0, 46.0]
FWHM = 15.9 16.6 17.5 mm mm mm; 15.9 16.6 17.5 {voxels}
Volume: 1610383 = 1610383 voxels = 329.2 resels
Voxel size: 1.0 1.0 1.0 mm mm mm; (resel = 4632.59 voxels)
Page 1

< >
1

Supplementary Fig 7a: PANK2>Controls

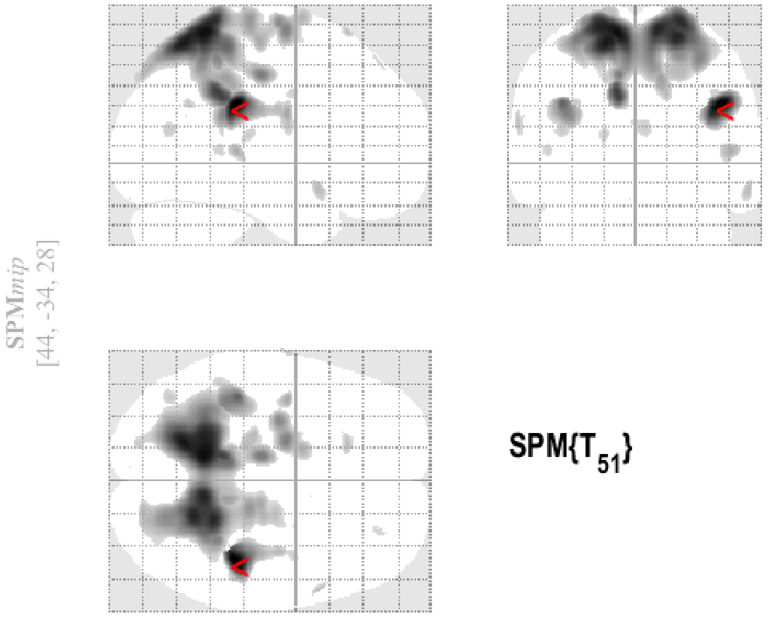

SPM{T_{51}}

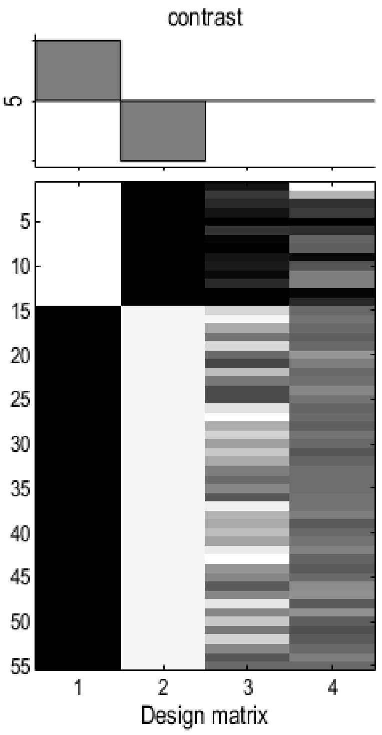

SPMresults: .\PKAN_vs_Controls

Height threshold T = 3.257890 {p<0.001 (unc.)}

Extent threshold k = 0 voxels

Statistics: *p-values adjusted for search volume*

set-level		cluster-level				peak-level					mm mm mm		
p	c	$p_{FWE-corr}$	$q_{FDR-corr}$	k_E	p_{uncorr}	$p_{FWE-corr}$	$q_{FDR-corr}$	T	$(Z_{\equiv})$	p_{uncorr}			
0.000	23	0.036	0.099	4176	0.009	0.000	0.001	7.18	5.94	0.000	44	-34	28
						0.596	0.416	3.93	3.66	0.000	39	-11	26
		0.000	0.000	56292	0.000	0.000	0.001	6.85	5.74	0.000	-12	-51	66
						0.000	0.001	6.64	5.61	0.000	-18	-62	61
						0.001	0.001	6.47	5.50	0.000	20	-57	63
		0.111	0.158	2746	0.027	0.017	0.021	5.35	4.74	0.000	-41	-33	29
		0.079	0.147	3167	0.019	0.067	0.064	4.88	4.40	0.000	-19	-7	67
						0.098	0.082	4.74	4.30	0.000	-29	-11	71
						0.472	0.348	4.07	3.77	0.000	-14	2	74
		0.797	0.982	397	0.371	0.375	0.283	4.19	3.87	0.000	-40	-7	23
		0.765	0.982	456	0.338	0.475	0.348	4.07	3.77	0.000	49	-33	4
		0.439	0.621	1142	0.135	0.617	0.417	3.91	3.64	0.000	-21	-25	68
						0.625	0.417	3.90	3.63	0.000	-16	-28	76
		0.933	0.982	127	0.629	0.649	0.420	3.88	3.61	0.000	59	10	-15
		0.934	0.982	123	0.635	0.683	0.440	3.84	3.58	0.000	-18	26	66
		0.915	0.982	167	0.574	0.756	0.494	3.75	3.51	0.000	-65	-6	19
		0.880	0.982	238	0.495	0.849	0.637	3.63	3.41	0.000	-41	-70	15
		0.935	0.982	121	0.639	0.857	0.637	3.62	3.40	0.000	-34	-59	29
		0.920	0.982	156	0.588	0.874	0.649	3.59	3.37	0.000	3	-40	17
		0.934	0.982	123	0.635	0.894	0.671	3.55	3.34	0.000	-19	-27	13

table shows 3 local maxima more than 8.0mm apart

Height threshold: T = 3.26, p = 0.001 (0.986)
Extent threshold: k = 0 voxels
Expected voxels per cluster, <k> = 535.507
Expected number of clusters, <c> = 4.29
FWEp: 4.987, FDRp: 5.316, FWEc: 4176, FDRc: 56292

Degrees of freedom = [1.0, 51.0]
FWHM = 16.3 17.4 18.4 mm mm mm; 16.3 17.4 18.4 {voxels}
Volume: 1628582 = 1628582 voxels = 296.0 resels
Voxel size: 1.0 1.0 1.0 mm mm mm; (resel = 5217.02 voxels)
Page 1

Supplementary Fig. 7b: PANK2<Controls

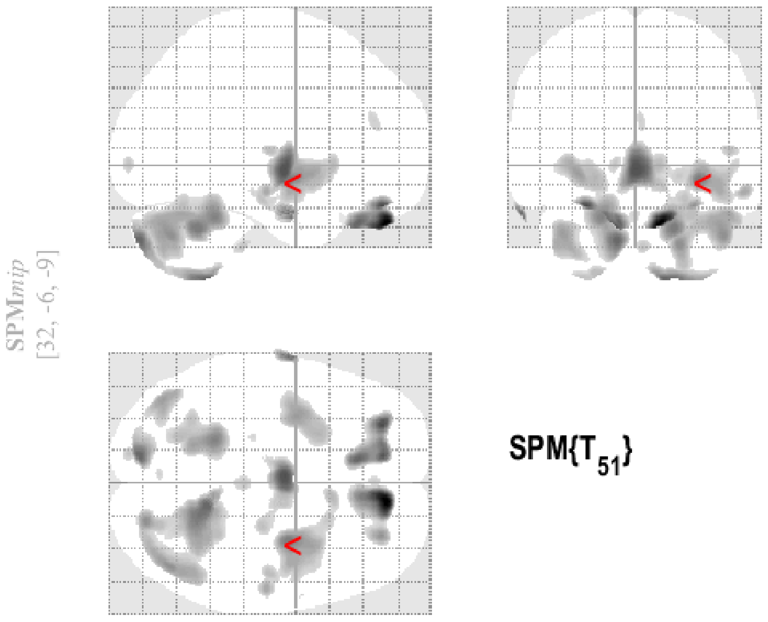

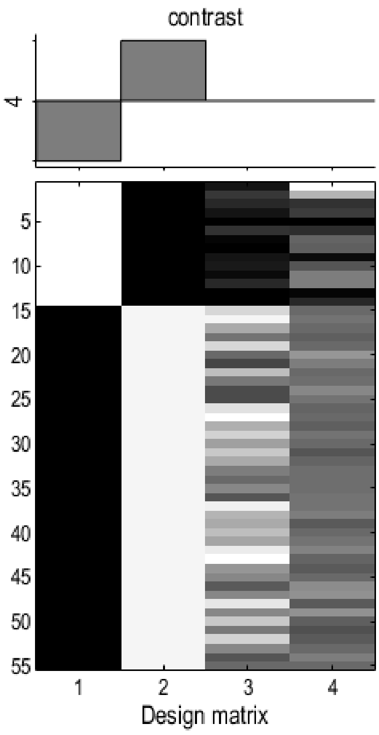

SPMresults: .\PKAN_vs_Controls
Height threshold T = 3.257890 {p<0.001 (unc.)}
Extent threshold k = 0 voxels

Statistics: *p-values adjusted for search volume*

set-level		cluster-level				peak-level					mm mm mm		
p	c	$p_{FWE-corr}$	$q_{FDR-corr}$	k_E	p_{uncorr}	$p_{FWE-corr}$	$q_{FDR-corr}$	T	$(Z_{\equiv})$	p_{uncorr}			
**0.000**	**23**	**0.122**	**0.096**	**2633**	**0.030**	**0.000**	**0.003**	**6.66**	**5.62**	**0.000**	**11**	**46**	**-32**
						0.278	0.225	4.33	3.98	0.000	29	44	-23
		**0.133**	**0.096**	**2525**	**0.033**	**0.013**	**0.055**	**5.45**	**4.82**	**0.000**	**-28**	**44**	**-23**
						0.019	0.056	5.31	4.72	0.000	-12	45	-34
						0.040	0.077	5.06	4.54	0.000	-10	31	-32
		**0.053**	**0.049**	**3678**	**0.013**	**0.017**	**0.056**	**5.35**	**4.74**	**0.000**	**-1**	**-8**	**-4**
						0.749	0.535	3.76	3.52	0.000	10	-10	-12
						0.945	0.771	3.44	3.25	0.001	-10	-18	-16
		**0.249**	**0.153**	**1795**	**0.067**	**0.045**	**0.077**	**5.02**	**4.50**	**0.000**	**-12**	**-86**	**-41**
		**0.002**	**0.009**	**8462**	**0.000**	**0.047**	**0.077**	**5.01**	**4.50**	**0.000**	**13**	**-49**	**-60**
						0.238	0.204	4.40	4.03	0.000	22	-82	-43
						0.259	0.216	4.36	4.00	0.000	45	-69	-38
		**0.901**	**0.746**	**196**	**0.539**	**0.064**	**0.096**	**4.90**	**4.41**	**0.000**	**-63**	**-6**	**-27**
		**0.003**	**0.009**	**7668**	**0.001**	**0.100**	**0.105**	**4.74**	**4.29**	**0.000**	**32**	**-6**	**-9**
						0.387	0.273	4.17	3.85	0.000	35	6	-6
						0.461	0.302	4.08	3.78	0.000	42	-4	-13
		**0.010**	**0.018**	**5999**	**0.002**	**0.104**	**0.105**	**4.72**	**4.28**	**0.000**	**19**	**-55**	**-34**
						0.765	0.535	3.74	3.50	0.000	28	-45	-27
						0.793	0.555	3.71	3.47	0.000	24	-63	-23
		**0.016**	**0.022**	**5304**	**0.004**	**0.154**	**0.138**	**4.57**	**4.16**	**0.000**	**-20**	**-46**	**-29**

table shows 3 local maxima more than 8.0mm apart

Height threshold: T = 3.26, p = 0.001 (0.986)
Extent threshold: k = 0 voxels
Expected voxels per cluster, <k> = 535.507
Expected number of clusters, <c> = 4.29
FWEp: 4.987, FDRp: 6.482, FWEc: 4108, FDRc: 3678

Degrees of freedom = [1.0, 51.0]
FWHM = 16.3 17.4 18.4 mm mm mm; 16.3 17.4 18.4 {voxels}
Volume: 1628582 = 1628582 voxels = 296.0 resels
Voxel size: 1.0 1.0 1.0 mm mm mm; (resel = 5217.02 voxels)
Page 1

< >

Supplementary Fig. 8a: CP-Kernicterus>Controls

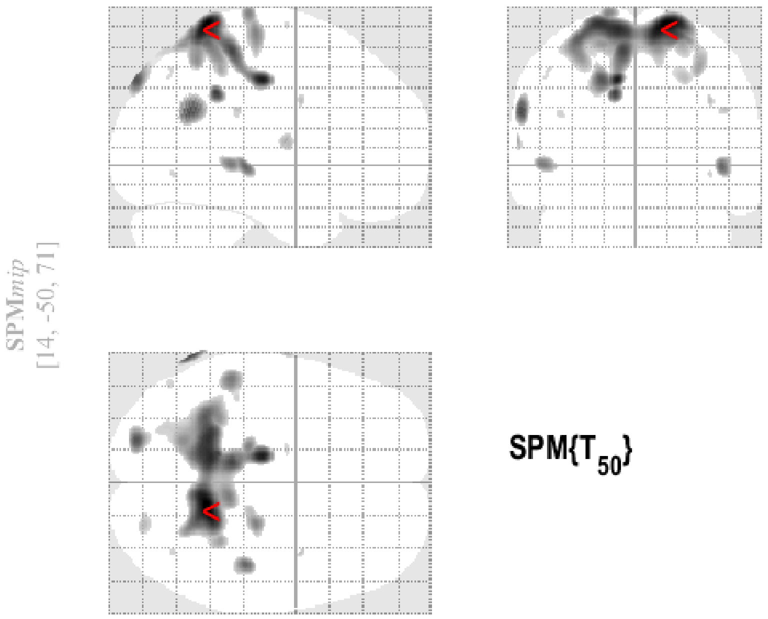

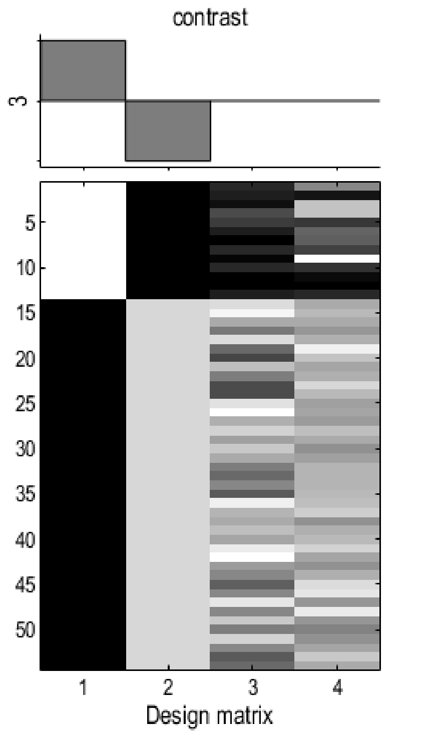

SPMresults: .\CP_Kernicterus_vs_Controls

Height threshold T = 3.261409 {p<0.001 (unc.)}

Extent threshold k = 0 voxels

Statistics: *p-values adjusted for search volume*

set-level		cluster-level				peak-level					mm mm mm		
p	c	$p_{FWE-corr}$	$q_{FDR-corr}$	k_E	p_{uncorr}	$p_{FWE-corr}$	$q_{FDR-corr}$	T	$(Z_{\equiv})$	p_{uncorr}			
0.000	16	0.000	0.000	22735	0.000	0.006	0.045	5.75	5.01	0.000	14	-50	71
						0.019	0.074	5.36	4.74	0.000	-12	-20	42
						0.031	0.075	5.20	4.63	0.000	-11	-48	68
		0.789	0.659	424	0.329	0.063	0.078	4.95	4.44	0.000	-13	-45	35
		0.779	0.659	441	0.320	0.085	0.078	4.84	4.36	0.000	-63	-59	25
		0.520	0.659	919	0.156	0.121	0.078	4.71	4.27	0.000	-20	-87	42
		0.731	0.659	524	0.278	0.185	0.098	4.54	4.14	0.000	44	-29	-3
		0.643	0.659	681	0.218	0.300	0.143	4.34	3.98	0.000	-52	-37	-1
		0.332	0.659	1396	0.086	0.362	0.157	4.25	3.91	0.000	23	-23	66
		0.734	0.659	519	0.281	0.365	0.157	4.25	3.91	0.000	-16	-27	77
		0.962	0.878	80	0.694	0.755	0.413	3.80	3.54	0.000	-65	-7	11
		0.884	0.812	251	0.457	0.871	0.558	3.64	3.41	0.000	24	-83	44
		0.977	0.878	37	0.804	0.898	0.602	3.59	3.37	0.000	38	1	31
		0.964	0.878	76	0.703	0.914	0.623	3.56	3.35	0.000	46	-34	27
		0.978	0.878	35	0.810	0.965	0.755	3.42	3.23	0.001	-42	-34	25
		0.984	0.878	17	0.878	0.965	0.755	3.42	3.23	0.001	-49	-69	47
		0.982	0.878	24	0.849	0.966	0.755	3.42	3.23	0.001	38	-68	-6
		0.978	0.878	34	0.813	0.978	0.835	3.36	3.18	0.001	-15	-7	71

table shows 3 local maxima more than 8.0mm apart

Height threshold: T = 3.26, p = 0.001 (0.991)
Extent threshold: k = 0 voxels
Expected voxels per cluster, <k> = 481.514
Expected number of clusters, <c> = 4.72
FWEp: 5.033, FDRp: 5.751, FWEc: 22735, FDRc: 22735

Degrees of freedom = [1.0, 50.0]
FWHM = 16.0 16.8 17.5 mm mm mm; 16.0 16.8 17.5 {voxels}
Volume: 1625174 = 1625174 voxels = 327.3 resels
Voxel size: 1.0 1.0 1.0 mm mm mm; (resel = 4704.82 voxels)

Supplementary Fig. 8b: CP-Kernicterus<Controls

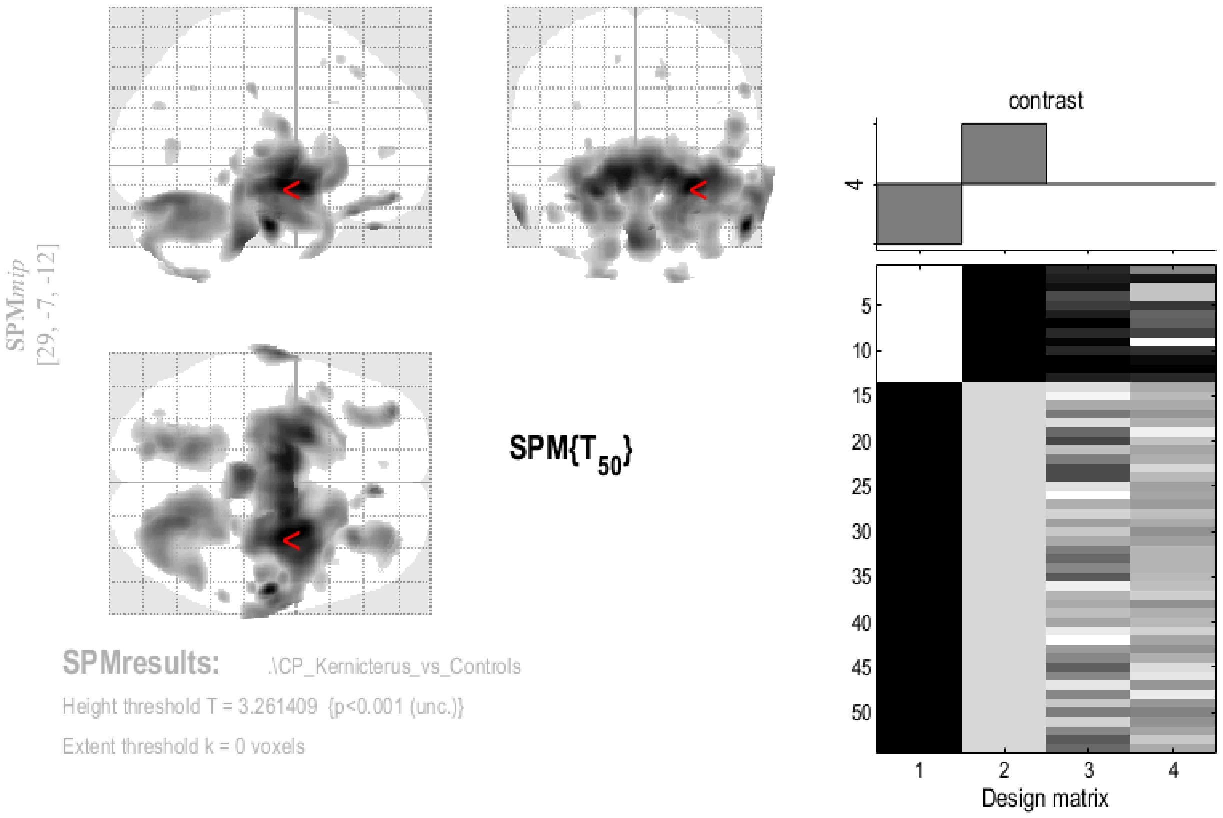

Statistics: ***p-values adjusted for search volume***

set-level		cluster-level				peak-level					
p	c	$p_{FWE-corr}$	$q_{FDR-corr}$	k_E	p_{uncorr}	$p_{FWE-corr}$	$q_{FDR-corr}$	T	$(Z_{\equiv})$	p_{uncorr}	mm mm mm
0.000	20	0.000	0.000	130625	0.000	0.000	0.002	6.75	5.67	0.000	29 -7 -12
						0.000	0.002	6.65	5.60	0.000	57 -16 -33
						0.000	0.002	6.65	5.60	0.000	27 1 -13
		0.263	0.431	1642	0.065	0.012	0.018	5.52	4.86	0.000	-65 -11 -27
						0.059	0.037	4.98	4.46	0.000	-67 -19 -26
						0.273	0.119	4.38	4.01	0.000	-63 -3 -26
		0.921	0.829	176	0.539	0.022	0.027	5.32	4.71	0.000	6 39 -35
		0.340	0.440	1372	0.088	0.051	0.036	5.03	4.50	0.000	-35 50 -19
						0.228	0.100	4.46	4.07	0.000	-31 36 -22
		0.000	0.000	14114	0.000	0.059	0.037	4.97	4.46	0.000	-15 -58 -27
						0.081	0.045	4.86	4.38	0.000	-18 -71 -20
						0.219	0.098	4.48	4.08	0.000	-16 -81 -45
		0.489	0.569	987	0.142	0.219	0.098	4.47	4.08	0.000	20 -57 -61
		0.909	0.829	201	0.509	0.407	0.168	4.19	3.86	0.000	56 -26 38
		0.741	0.829	506	0.287	0.473	0.184	4.12	3.80	0.000	-47 13 -27
		0.883	0.829	254	0.454	0.515	0.200	4.07	3.76	0.000	61 -41 -8
		0.859	0.829	299	0.415	0.687	0.300	3.88	3.61	0.000	-27 -68 9
		0.909	0.829	201	0.509	0.697	0.304	3.87	3.60	0.000	12 31 46
		0.872	0.829	275	0.435	0.759	0.354	3.79	3.54	0.000	-42 8 -47
						0.981	0.891	3.35	3.16	0.001	-34 3 -50

table shows 3 local maxima more than 8.0mm apart

Height threshold: T = 3.26, p = 0.001 (0.991)
Extent threshold: k = 0 voxels
Expected voxels per cluster, <k> = 481.514
Expected number of clusters, <c> = 4.72
FWEp: 5.033, FDRp: 4.806, FWEc: 14114, FDRc: 14114

Degrees of freedom = [1.0, 50.0]
FWHM = 16.0 16.8 17.5 mm mm mm; 16.0 16.8 17.5 {voxels}
Volume: 1625174 = 1625174 voxels = 327.3 resels
Voxel size: 1.0 1.0 1.0 mm mm mm; (resel = 4704.82 voxels)
Page 1

< >

Supplementary Fig. 9a: CP-Preterm>Controls

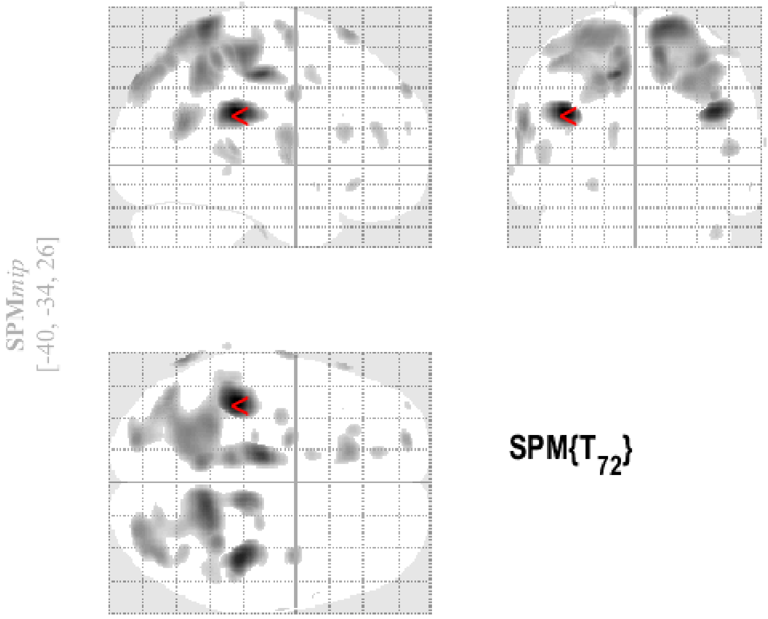

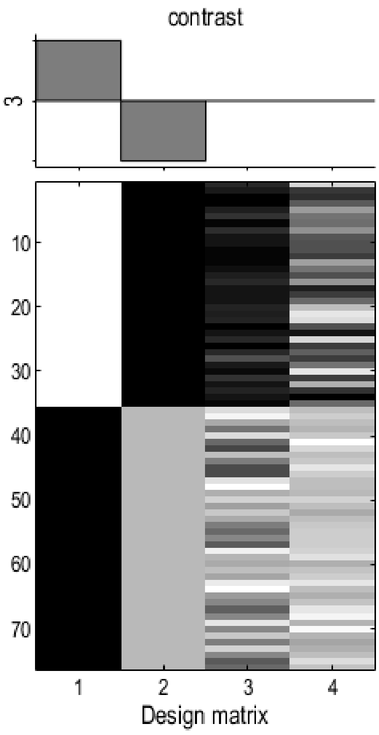

SPMresults: .\CP_Preterm_vs_Controls

Height threshold T = 3.207326 {p<0.001 (unc.)}

Extent threshold k = 0 voxels

Statistics: *p-values adjusted for search volume*

set-level		cluster-level				peak-level							
p	c	$p_{FWE-corr}$	$q_{FDR-corr}$	k_E	p_{uncorr}	$p_{FWE-corr}$	$q_{FDR-corr}$	T	$(Z_{\equiv})$	p_{uncorr}	mm	mm	mm
0.000	28	0.032	0.065	3774	0.007	0.000	0.000	7.56	6.46	0.000	-40	-34	26
		0.067	0.102	2964	0.015	0.000	0.000	6.79	5.95	0.000	41	-32	26
		0.000	0.000	21780	0.000	0.000	0.002	6.22	5.54	0.000	-12	-21	45
						0.015	0.033	5.21	4.78	0.000	-9	-53	47
						0.033	0.045	4.98	4.60	0.000	-28	-48	66
		0.000	0.000	19176	0.000	0.001	0.002	6.11	5.46	0.000	14	-50	70
						0.005	0.013	5.54	5.04	0.000	11	-30	56
						0.037	0.045	4.94	4.57	0.000	33	-44	53
		0.571	0.725	769	0.179	0.028	0.045	5.02	4.64	0.000	-62	-62	20
		0.588	0.725	739	0.187	0.111	0.085	4.58	4.28	0.000	-14	27	66
		0.775	0.743	423	0.315	0.146	0.087	4.49	4.20	0.000	40	-4	34
		0.608	0.725	703	0.198	0.166	0.094	4.44	4.16	0.000	-20	-6	65
		0.877	0.744	251	0.443	0.218	0.112	4.34	4.08	0.000	-20	58	37
		0.750	0.743	464	0.293	0.223	0.112	4.33	4.07	0.000	-65	-6	12
						0.327	0.171	4.18	3.94	0.000	-65	-6	1
		0.824	0.743	342	0.367	0.359	0.184	4.13	3.90	0.000	49	-35	6
		0.885	0.744	235	0.458	0.447	0.232	4.03	3.82	0.000	-33	-10	70
		0.908	0.744	193	0.505	0.464	0.234	4.02	3.80	0.000	-59	24	15
		0.709	0.743	530	0.261	0.586	0.300	3.89	3.69	0.000	38	-62	21
		0.827	0.743	336	0.372	0.608	0.310	3.87	3.67	0.000	-13	42	16

table shows 3 local maxima more than 8.0mm apart

Height threshold: T = 3.21, p = 0.001 (0.991)
Extent threshold: k = 0 voxels
Expected voxels per cluster, <k> = 453.409
Expected number of clusters, <c> = 4.73
FWEp: 4.843, FDRp: 4.934, FWEc: 3774, FDRc: 19176

Degrees of freedom = [1.0, 72.0]
FWHM = 15.4 16.3 16.8 mm mm mm; 15.4 16.3 16.8 {voxels}
Volume: 1500417 = 1500417 voxels = 334.2 resels
Voxel size: 1.0 1.0 1.0 mm mm mm; (resel = 4233.06 voxels)
Page 1

< >

Supplementary Fig. 9b: CP-Preterm<Controls

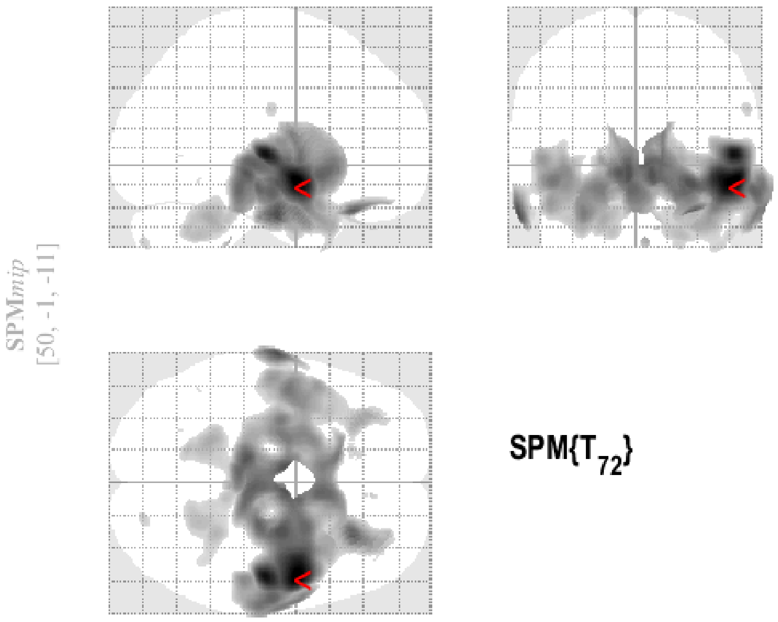

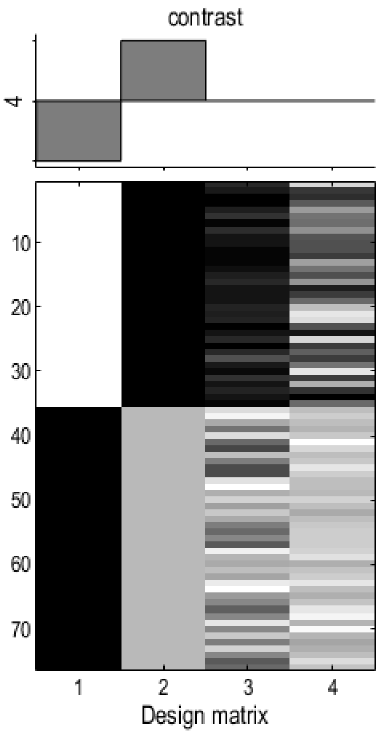

SPMresults: .\CP_Preterm_vs_Controls

Height threshold T = 3.207326 {p<0.001 (unc.)}

Extent threshold k = 0 voxels

Statistics: ***p-values adjusted for search volume***

set-level		cluster-level				peak-level					mm mm mm
p	c	$p_{FWE-corr}$	$q_{FDR-corr}$	k_E	p_{uncorr}	$p_{FWE-corr}$	$q_{FDR-corr}$	T	$(Z_{\equiv})$	p_{uncorr}	
0.023	10	0.000	0.000	127051	0.000	0.000	0.000	8.68	7.16	0.000	50 -1 -11
						0.000	0.000	8.15	6.84	0.000	50 -19 4
						0.000	0.000	6.62	5.83	0.000	-64 -11 -28
		0.979	0.980	32	0.813	0.143	0.053	4.49	4.20	0.000	4 -37 -40
		0.987	0.980	9	0.915	0.758	0.387	3.71	3.54	0.000	8 38 -35
		0.931	0.980	146	0.567	0.758	0.387	3.71	3.54	0.000	58 -16 27
		0.972	0.980	49	0.760	0.783	0.412	3.68	3.51	0.000	-13 29 -31
		0.931	0.980	146	0.567	0.827	0.456	3.63	3.46	0.000	21 -83 -42
		0.988	0.980	7	0.928	0.969	0.777	3.34	3.21	0.001	9 34 -34
		0.990	0.980	1	0.980	0.974	0.795	3.32	3.20	0.001	-10 34 -34
		0.987	0.980	8	0.921	0.984	0.896	3.26	3.14	0.001	-46 -54 -10
		0.989	0.980	3	0.958	0.987	0.925	3.25	3.13	0.001	-25 -64 3

table shows 3 local maxima more than 8.0mm apart

Height threshold: T = 3.21, p = 0.001 (0.991)
Extent threshold: k = 0 voxels
Expected voxels per cluster, <k> = 453.409
Expected number of clusters, <c> = 4.73
FWEp: 4.843, FDRp: 4.526, FWEc: 127051, FDRc: 127051

Degrees of freedom = [1.0, 72.0]
FWHM = 15.4 16.3 16.8 mm mm mm; 15.4 16.3 16.8 {voxels}
Volume: 1500417 = 1500417 voxels = 334.2 resels
Voxel size: 1.0 1.0 1.0 mm mm mm; (resel = 4233.06 voxels)

Supplementary Fig. 10a: CP-Term (HIE)>Controls

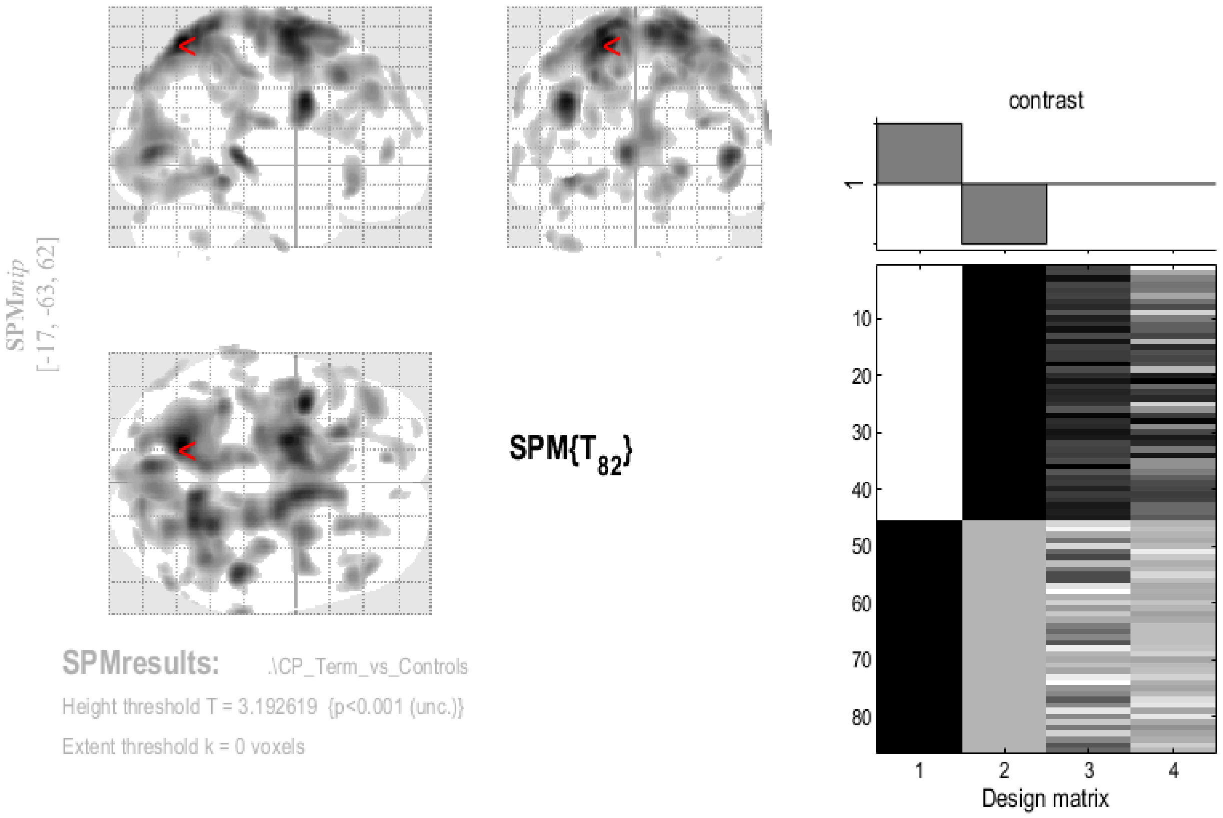

Statistics: ***p-values adjusted for search volume***

set-level		cluster-level				peak-level							
p	c	$p_{FWE-corr}$	$q_{FDR-corr}$	k_E	p_{uncorr}	$p_{FWE-corr}$	$q_{FDR-corr}$	T	$(Z_{\equiv})$	p_{uncorr}	mm	mm	mm
0.000	39	0.000	0.000	144894	0.000	0.000	0.001	6.72	5.98	0.000	-17	-63	62
						0.000	0.002	6.37	5.73	0.000	-39	3	31
						0.000	0.002	6.31	5.68	0.000	-20	-6	66
		0.178	0.349	1928	0.040	0.001	0.004	5.83	5.31	0.000	49	-33	3
		0.046	0.188	3342	0.010	0.018	0.028	5.11	4.75	0.000	-37	-41	-10
						0.409	0.223	4.06	3.86	0.000	-23	-46	-9
						0.506	0.271	3.95	3.77	0.000	-44	-43	-19
		0.312	0.349	1376	0.077	0.090	0.067	4.62	4.34	0.000	35	-19	20
		0.574	0.524	770	0.175	0.124	0.084	4.51	4.25	0.000	69	-31	23
		0.354	0.349	1254	0.089	0.145	0.095	4.46	4.21	0.000	50	29	-10
		0.547	0.524	821	0.162	0.185	0.115	4.37	4.13	0.000	-27	28	-14
		0.186	0.349	1882	0.042	0.201	0.121	4.34	4.11	0.000	45	38	37
						0.620	0.330	3.84	3.67	0.000	53	30	32
						0.806	0.438	3.64	3.50	0.000	42	26	47
		0.351	0.349	1261	0.089	0.201	0.121	4.34	4.11	0.000	-42	-29	23
		0.264	0.349	1539	0.063	0.211	0.125	4.32	4.09	0.000	54	-70	13
						0.578	0.311	3.88	3.71	0.000	50	-60	-1
		0.723	0.683	517	0.263	0.238	0.134	4.28	4.05	0.000	-11	47	5
		0.702	0.683	551	0.248	0.407	0.223	4.06	3.86	0.000	-38	-12	-42
		0.295	0.349	1432	0.072	0.471	0.251	3.99	3.80	0.000	-66	-36	28

table shows 3 local maxima more than 8.0mm apart

Height threshold: T = 3.19, p = 0.001 (0.992)
Extent threshold: k = 0 voxels
Expected voxels per cluster, <k> = 444.357
Expected number of clusters, <c> = 4.88
FWEp: 4.803, FDRp: 4.893, FWEc: 3342, FDRc: 144894

Degrees of freedom = [1.0, 82.0]
FWHM = 15.3 16.1 16.6 mm mm mm; 15.3 16.1 16.6 {voxels}
Volume: 1516170 = 1516170 voxels = 348.7 resels
Voxel size: 1.0 1.0 1.0 mm mm mm; (resel = 4096.99 voxels)
Page 1

Supplementary Fig. 10b: CP-Term (HIE)<Controls

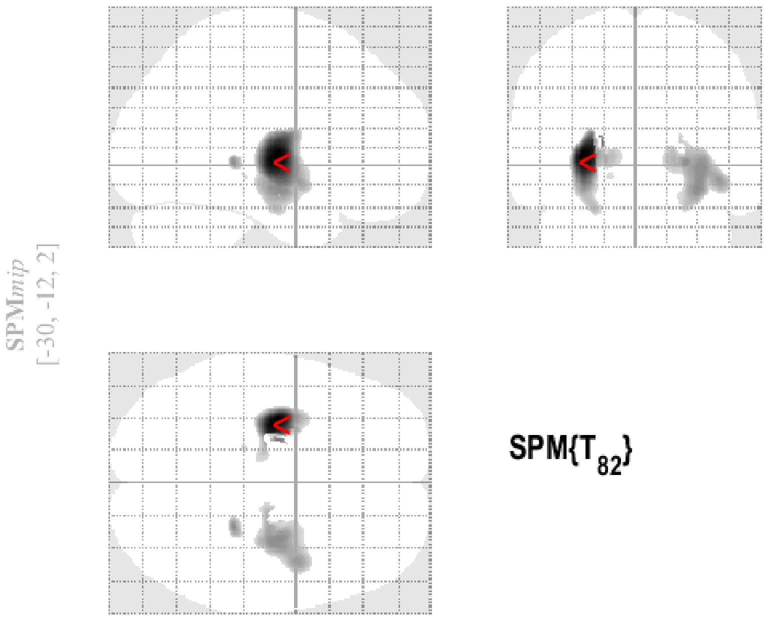

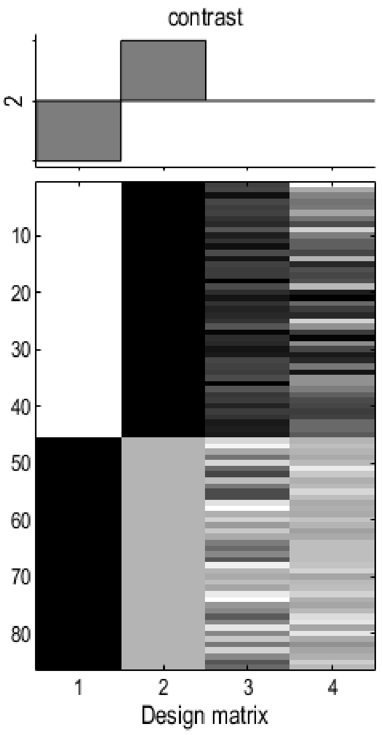

SPMresults: .\CP_Term_vs_Controls
Height threshold T = 3.192619 {p<0.001 (unc.)}
Extent threshold k = 0 voxels

Statistics: ***p-values adjusted for search volume***

set-level		cluster-level				peak-level					mm mm mm		
p	c	$p_{FWE-corr}$	$q_{FDR-corr}$	k_E	p_{uncorr}	$p_{FWE-corr}$	$q_{FDR-corr}$	T	$(Z_{\equiv})$	p_{uncorr}			
0.539	5	0.001	0.001	8149	0.000	0.000	0.000	7.22	6.33	0.000	-30	-12	2
						0.287	0.116	4.21	3.99	0.000	-18	-18	1
						0.583	0.224	3.88	3.70	0.000	-24	-13	-24
		0.984	0.850	23	0.845	0.030	0.047	4.96	4.62	0.000	-20	-12	10
		0.001	0.001	8406	0.000	0.085	0.059	4.64	4.36	0.000	31	-11	-14
						0.108	0.059	4.56	4.29	0.000	33	-14	-4
						0.184	0.089	4.37	4.13	0.000	43	-1	-10
		0.820	0.585	358	0.351	0.092	0.059	4.61	4.34	0.000	25	-35	-1
		0.984	0.850	22	0.850	0.985	0.856	3.26	3.15	0.001	-16	-30	-8

table shows 3 local maxima more than 8.0mm apart

Height threshold: T = 3.19, p = 0.001 (0.992)
Extent threshold: k = 0 voxels
Expected voxels per cluster, <k> = 444.357
Expected number of clusters, <c> = 4.88
FWEp: 4.803, FDRp: 4.958, FWEc: 8149, FDRc: 8149

Degrees of freedom = [1.0, 82.0]
FWHM = 15.3 16.1 16.6 mm mm mm; 15.3 16.1 16.6 {voxels}
Volume: 1516170 = 1516170 voxels = 348.7 resels
Voxel size: 1.0 1.0 1.0 mm mm mm; (resel = 4096.99 voxels)

Metabolic patterns in Brain [^{18}F]fluorodeoxyglucose PET relate to aetiology in paediatric dystonia

Tsagkaris S, Yau EKC, McClelland VM, Papandreou A, Siddiqui A, Lumsden DE, Kaminska M, Guedj E, Hammers A & Lin JP

Data and modified figures from *Brain* 2022; issue and page numbers to be confirmed

Inherited dystonias exhibiting regional hypometabolism when compared to controls

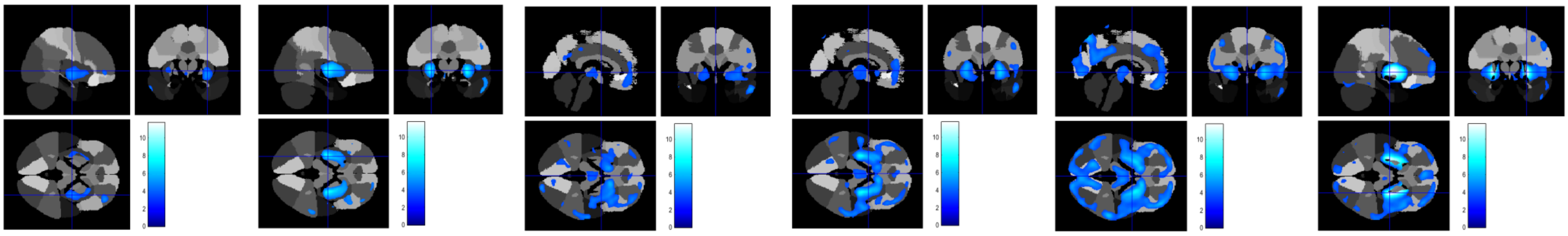

FDG uptake compared to controls in inherited dystonias that only showed hypometabolism (i.e. excluding PANK2). Areas of relative regional hypometabolism are displayed in a blue-scale with brighter tones indicating higher t-scores. From left to right: TOR1A; Putamen-frontal hypometabolism. THAP1; Caudate-putaminal and right fronto-parietal hypometabolism. SGCE; Head of caudate, globus pallidus, inferior frontal lobe hypometabolism. KMT2B; Marked caudate-putaminal (striatal) and bi-frontal and bi-parietal hypometabolism. HPRT1; Extreme caudate-putaminal-pallidal, medial thalamic, and pan-hemispheric and antero-superior cerebellar hypometabolism. GCDH; Relative regional hypometabolism in the posterior putamina and globi pallidi. All results are displayed on the 97-region version of the maximum probability atlas derived from the Hammers Atlas Database (www.brain-development.org/brain-atlases), which is a three-dimensional maximum probability atlas of the human brain.

Pantothenate kinase-associated neurodegeneration (PKAN; PANK2 gene mutation)

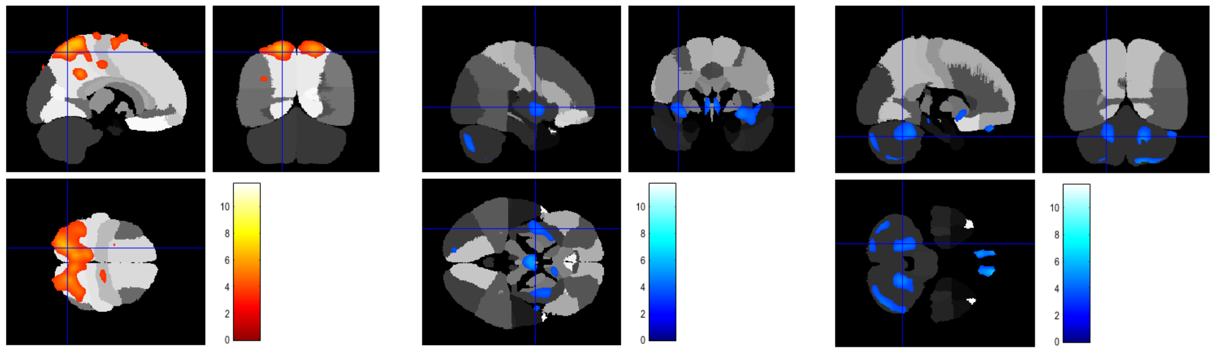

FDG uptake in PANK2 cases compared to controls; Left: Areas of regional relative hypermetabolism seen in the superior parietal lobes and displayed in a yellow-scale with brighter tones indicating higher t-scores. Middle and right: Hypometabolic areas noted in the peri-insular cortex, cerebellar dentate nuclei and posterior inferior cerebellar cortex

Cerebral Palsy (CP)

CP - Kernicterus

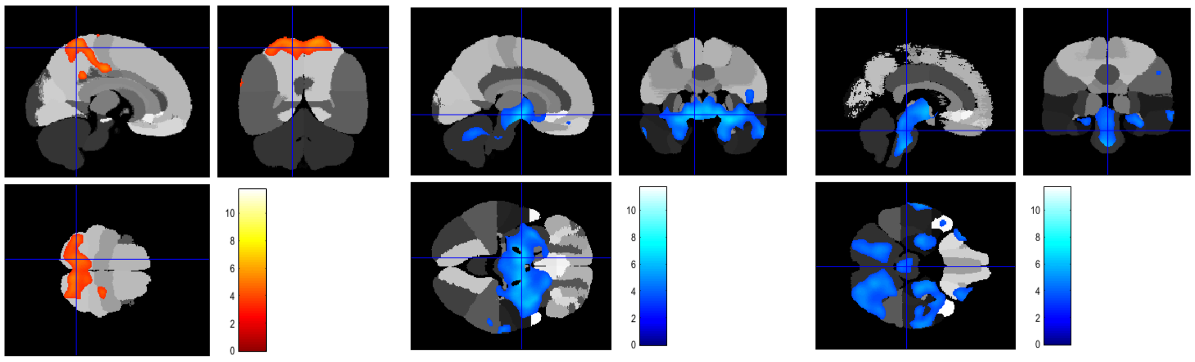

FDG uptake in CP-kernicterus cases compared to controls. Left: Relative hypermetabolism in superior parietal lobes. Middle and right: Areas of relative hypometabolism, including the antero-medial temporal cortex, globi pallidi, thalami, midbrain, pons, dentate-cortical cerebellum and peri-insular cortex.

CP - Preterm

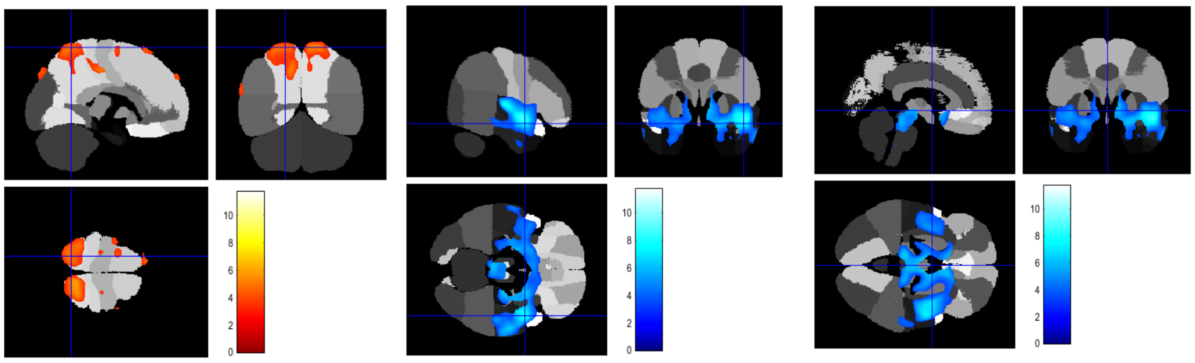

FDG uptake in CP-preterm cases compared to controls. Left: Relative hypermetabolism in the superior parietal. Middle and right: Areas of relative hypometabolism, involving the superior-medial temporal lobes, peri-insular cortex, globi pallidi, thalami, brainstem.

CP - Term

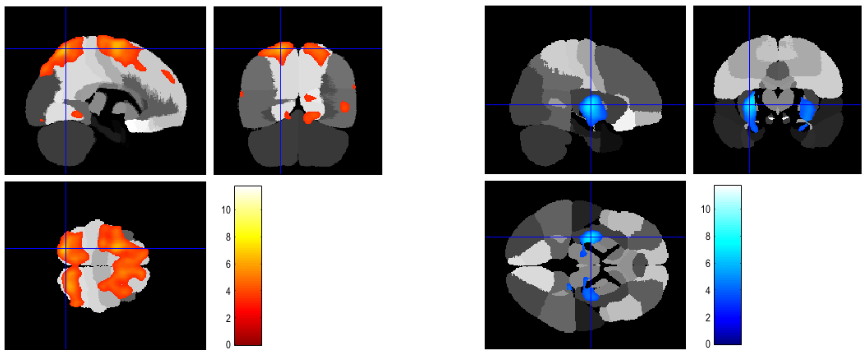

FDG uptake in CP-term cases compared to controls. Left: Regionally increased uptake in the superior anterior frontal and superior parietal lobules. Right: Regionally reduced uptake in the posterior putamina, globi pallidi and lateral parts of the thalami.

Evelina London Children's Hospital
King's College London
King's Health Partners
PET Centre
NHS Guy's and St Thomas' NHS Foundation Trust
